# Supplementary material for: Deprescribing montelukast in children with asthma: a systematic review
Source: BMJ Open. 2022 Jan 31;12(1):e053112. doi: 10.1136/bmjopen-2021-053112 (PMC8804657; doi:10.1136/bmjopen-2021-053112)
Supplement: Supplementary data [file bmjopen-2021-053112supp001.pdf]

Deprescribing Montelukast in Children with Asthma: A Systematic Review (SUPPLEMENTARY DATA SECTION)

Eleanor Dixon[1,2], Charlotte King[3], Andrew Lilley[4], Ian Sinha[5], Daniel B Hawcutt[2,4]

1: Department of Pharmacology and Therapeutics, University of Liverpool, UK

2: Department of Women's and Children's Health, University of Liverpool, UK

3: Liverpool University Hospital Trust, Liverpool, UK

4: NIHR Alder Hey Clinical Research Facility, Alder Hey Children's Hospital, Liverpool, UK

5: Department of Respiratory Medicine, Alder Hey Children's Hospital, Liverpool, UK

Tables: 3

Keywords: Montelukast, Adverse Drug Reactions, Asthma, Paediatric, Systematic Review

Methods

Table S1: Search Strategy

Strategy 918451 #	Database	Search term	Results
1	EMBASE	CHILD/ OR INFANT/ OR BABY/ OR NEWBORN/	2417196
2	EMBASE	JUVENILE/ OR CHILD/ OR BOY/ OR GIRL/ OR INFANT/ OR TODDLER/ OR "SCHOOL CHILD"/ OR "PRESCHOOL CHILD"/	2301714
3	EMBASE	JUVENILE/ OR ADOLESCENT/	1563364
4	EMBASE	PEDIATRICS/	78300
5	EMBASE	((Paediatric* OR Infan* OR Newborn* OR Bab* OR Neonat* OR Preterm* OR Prematur* OR postmatur* OR Child* OR School* OR "Nursery school*" OR Kindergar* OR "Primary school*" OR "Secondary school*" OR "Elementary school*" OR "High school*" OR Highschool* OR Preschool* OR Kid* OR Toddler* OR Adoles* OR Teen* OR Boy* OR Girl* Minor* OR Pubert* OR Pubescen* OR Prepubescen*) NOT REVIEW*).ti,ab	3217377
6	EMBASE	"MINOR (PERSON)"/	653
7	EMBASE	ADOLESCENCE/ OR PUBERTY/ OR PREPUBERTY/	70704
8	EMBASE	(1 OR 2 OR 3 OR 4 OR 5 OR 6 OR 7)	4839396
9	EMBASE	8 [Humans]	4025357
10	EMBASE	ASTHMA/ OR "ASTHMA BRONCHIALE"/	231772
11	EMBASE	((Asthma* OR Wheez* OR "Bronchial asthma*" OR "stable asthma") NOT REVIEW).ti,ab	216313
12	EMBASE	(10 OR 11)	284696
13	EMBASE	12 [Humans]	238534
14	EMBASE	DEPRESCRIPTION/ OR DEPRESCRIPTIONS/	501
15	EMBASE	"TREATMENT WITHDRAWAL"/ OR "DRUG WITHDRAWAL"/	215067
16	EMBASE	((deprescrib* OR stop* OR withdraw* OR ceas* OR discontinu* OR de-prescrib*) NOT REVIEW).ti,ab	536525
17	EMBASE	(14 OR 15 OR 16)	680842
18	EMBASE	17 [Humans]	519666
19	EMBASE	MONTELUKAST/	9658
20	EMBASE	((montelukast OR singular) NOT REVIEW).ti,ab	15377
21	EMBASE	(19 OR 20)	21572
22	EMBASE	21 [Humans]	14849

23	EMBASE	(8 AND 12 AND 17 AND 21)	194
24	EMBASE	23 [Humans]	191
25	Medline	CHILD/ OR "CHILD, PRESCHOOL"/	1916435
26	Medline	ADOLESCENT/ OR MINORS/	2036089
27	Medline	INFANT/ OR "INFANT, NEWBORN"/	1139739
28	Medline	((Paediatric* OR Infan* OR Newborn* OR Bab* OR Neonat* OR Preterm* OR Prematur* OR postmatur* OR Child* OR School* OR "Nursery school*" OR Kindergar* OR "Primary school*" OR "Secondary school*" OR "Elementary school*" OR "High school*" OR Highschool* OR Preschool* OR Kid* OR Toddler* OR Adoles* OR Teen* OR Boy* OR Girl* Minor* OR Pubert* OR Pubescen* OR Prepubescen*) NOT REVIEW*).ti,ab	2716250
29	Medline	PUBERTY/	13325
30	Medline	(25 OR 26 OR 27 OR 28 OR 29)	4733651
31	Medline	ASTHMA/	125895
32	Medline	((Asthma* OR Wheez* OR "Bronchial asthma*" OR "stable asthma") NOT REVIEW).ti,ab	155279
33	Medline	(31 OR 32)	177833
34	Medline	DEPRESCRIPTIONS/	466
35	Medline	((deprescrib* OR stop* OR withdraw* OR ceas* OR discontinu* OR de-prescrib*) NOT REVIEW).ti,ab	369364
36	Medline	(34 OR 35)	369497
37	Medline	((montelukast OR singular) NOT REVIEW).ti,ab	13162
38	Medline	(30 AND 33 AND 36 AND 37)	54
39	Medline	38 [Humans]	50
40	PubMed	((Paediatric* OR Infan* OR Newborn* OR Bab* OR Neonat* OR Preterm* OR Prematur* OR postmatur* OR Child* OR School* OR "Nursery school*" OR Kindergar* OR "Primary school*" OR "Secondary school*" OR "Elementary school*" OR "High school*" OR Highschool* OR Preschool* OR Kid* OR Toddler* OR Adoles* OR Teen* OR Boy* OR Girl* Minor* OR Pubert* OR Pubescen* OR Prepubescen*) NOT REVIEW*).ti,ab	143769
41	PubMed	((Asthma* OR Wheez* OR "Bronchial asthma*" OR "stable asthma") NOT REVIEW).ti,ab	161822
42	PubMed	((deprescrib* OR stop* OR withdraw* OR ceas* OR discontinu* OR de-prescrib*) NOT REVIEW).ti,ab	361553
43	PubMed	((montelukast OR singular) NOT REVIEW).ti,ab	13080
44	PubMed	(40 AND 41 AND 42 AND 43)	0
45	CINAHL	CHILD/ OR INFANT/ OR ADOLESCENCE/ OR "MINORS (LEGAL)"/ OR "CHILD, PRESCHOOL"/	892377
46	CINAHL	PEDIATRICS/ OR NEONATOLOGY/	20791
47	CINAHL	PUBERTY/	3060
48	CINAHL	ADOLESCENCE/ OR CHILD/ OR "MINORS (LEGAL)"/	778755
49	CINAHL	((Paediatric* OR Infan* OR Newborn* OR Bab* OR Neonat* OR Preterm* OR Prematur* OR postmatur* OR Child* OR School* OR "Nursery school*" OR Kindergar* OR "Primary school*" OR "Secondary school*" OR "Elementary school*" OR "High school*" OR Highschool* OR Preschool* OR Kid* OR Toddler* OR Adoles* OR Teen* OR Boy* OR Girl* Minor* OR Pubert* OR Pubescen* OR Prepubescen*) NOT REVIEW*).ti,ab	841196
50	CINAHL	(45 OR 46 OR 47 OR 48 OR 49)	1279403

51	CINAHL	ASTHMA/	34317
52	CINAHL	((Asthma* OR Wheez* OR "Bronchial asthma*" OR "stable asthma") NOT REVIEW).ti,ab	36601
53	CINAHL	((deprescrib* OR stop* OR withdraw* OR ceas* OR discontinu* OR de-prescrib*) NOT REVIEW).ti,ab	73381
54	CINAHL	(51 OR 52)	45034
55	CINAHL	MONTELUKAST/	368
56	CINAHL	((montelukast OR singular) NOT REVIEW).ti,ab	1953
57	CINAHL	(55 OR 56)	2122
58	CINAHL	(50 AND 53 AND 54 AND 57)	14

Results

Table S2: Results of assessment of risk bias of randomised control trials| The Cochrane Collaboration's tool was used. Green= low risk of bias, Yellow= unclear risk of bias, Red= high risk of bias.

	**Kim *et al.*, 2020**	**Montuschi *et al.*, 2007**	**Kim *et al.*, 2005**
Random sequence generation (randomisation bias)	Green	Yellow	Yellow
Allocation concealment (randomisation bias)	Green	Green	Green
Baseline differences (randomisation bias)	Green	Green	Green
Participant awareness (deviation bias)	Green	Green	Green
Deliverer awareness (deviation bias)	Green	Green	Green
Appropriate analysis (deviation bias)	Green	Green	Green
Available data (data bias)	Green	Green	Green
Appropriate measure (measuring bias)	Green	Green	Green
Difference of measure (measuring bias)	Green	Green	Green
Assessor awareness (measuring bias)	Green	Green	Green
Pre-specified data analysis (reporting bias)	Green	Yellow	Green
Numerical analysis (reporting bias)	Green	Green	Green
Other bias	Green	Green	Green

Table S3: Results of assessment of risk bias of case-controlled and cohort studies| The Newcastle-Ottawa Quality Assessment Form was used. Green= star (low risk of bias), Yellow= unknown risk of bias, Red= No star (high risk of bias).

	**Lee *et al.*, 2005**	**Bratton *et al.*, 1999**
Representativeness of the exposed cohort (selection bias)	Green	Green
Selection of the non-exposed cohort (selection bias)	Red	Green
Ascertainment of exposure (selection bias)	Green	Green
Outcome of interest stated (selection bias)	Green	Green
Comparability bias	Red	Green
Assessment of outcome (outcome bias)	Green	Green
Appropriate follow-up of outcomes (outcome bias)	Yellow	Yellow
Appropriate follow-up of patients (outcome bias)	Green	Green
**Overall quality**	Green	Green
