# Supplementary material for: Deprescribing montelukast in children with asthma: a systematic review
Source: BMJ Open. 2022 Jan 31;12(1):e053112. doi: 10.1136/bmjopen-2021-053112 (PMC8804657; doi:10.1136/bmjopen-2021-053112)
Supplement: Author's manuscript [file bmjopen-2021-053112.draft_revisions.pdf]

BMJ Open is committed to open peer review. As part of this commitment we make the peer review history of every article we publish publicly available.

When an article is published we post the peer reviewers' comments and the authors' responses online. We also post the versions of the paper that were used during peer review. These are the versions that the peer review comments apply to.

The versions of the paper that follow are the versions that were submitted during the peer review process. They are not the versions of record or the final published versions. They should not be cited or distributed as the published version of this manuscript.

BMJ Open is an open access journal and the full, final, typeset and author-corrected version of record of the manuscript is available on our site with no access controls, subscription charges or pay-per-view fees (http://bmjopen.bmj.com).

If you have any questions on BMJ Open's open peer review process please email info.bmjopen@bmj.com

BMJ Open

Deprescribing Montelukast in Children with Asthma: A Systematic Review

Journal:	*BMJ Open*
Manuscript ID	bmjopen-2021-053112
Article Type:	Original research
Date Submitted by the Author:	05-May-2021
Complete List of Authors:	Dixon, Eleanor; University of Liverpool Faculty of Health and Life Sciences, Department of Pharmacology and Therapeutics; University of Liverpool, Department of Women's and Children's Health<br>King, Charlotte; Liverpool University Hospitals NHS Foundation Trust, Royal Liverpool University Hospital<br>Lilley, Andrew; Alder Hey Children's NHS Foundation Trust, Department of Respiratory Medicine<br>Sinha, Ian; Alder Hey Children's NHS Foundation Trust<br>Hawcutt, Daniel; University of Liverpool, Department of Women's and Children's Health; Alder Hey Children's Hospital Clinical Research Facility
Keywords:	Asthma < THORACIC MEDICINE, CLINICAL PHARMACOLOGY, PAEDIATRICS

SCHOLARONE™
Manuscripts

I, the Submitting Author has the right to grant and does grant on behalf of all authors of the Work (as defined in the below author licence), an exclusive licence and/or a non-exclusive licence for contributions from authors who are: i) UK Crown employees; ii) where BMJ has agreed a CC-BY licence shall apply, and/or iii) in accordance with the terms applicable for US Federal Government officers or employees acting as part of their official duties; on a worldwide, perpetual, irrevocable, royalty-free basis to BMJ Publishing Group Ltd ("BMJ") its licensees and where the relevant Journal is co-owned by BMJ to the co-owners of the Journal, to publish the Work in this journal and any other BMJ products and to exploit all rights, as set out in our licence.

The Submitting Author accepts and understands that any supply made under these terms is made by BMJ to the Submitting Author unless you are acting as an employee on behalf of your employer or a postgraduate student of an affiliated institution which is paying any applicable article publishing charge ("APC") for Open Access articles. Where the Submitting Author wishes to make the Work available on an Open Access basis (and intends to pay the relevant APC), the terms of reuse of such Open Access shall be governed by a Creative Commons licence – details of these licences and which Creative Commons licence will apply to this Work are set out in our licence referred to above.

Other than as permitted in any relevant BMJ Author's Self Archiving Policies, I confirm this Work has not been accepted for publication elsewhere, is not being considered for publication elsewhere and does not duplicate material already published. I confirm all authors consent to publication of this Work and authorise the granting of this licence.

For peer review only

Deprescribing Montelukast in Children with Asthma: A Systematic Review

Eleanor G. Dixon[1,2], Charlotte King[3], Andrew Lilley[4], Ian Sinha[4], Daniel B Hawcutt[2,5],

1: Department of Pharmacology and Therapeutics, University of Liverpool, UK

2: Department of Women's and Children's Health, University of Liverpool, UK

3: Liverpool University Hospital Trust, Liverpool, UK

4: Department of Respiratory Medicine, Alder Hey Children's Hospital, Liverpool, UK

5: NIHR Alder Hey Clinical Research Facility, Alder Hey Children's Hospital, Liverpool, UK

Corresponding Author:

Dr. Dan Hawcutt
Alder Hey Children's Hospital
East Prescot Road
Liverpool
L14 5AB
Email: dhawcutt@liverpool.ac.uk

Word Count: 2447

Tables: 4

Figures: 1

Keywords: Montelukast, Deprescribing, Asthma, Paediatric, Systematic Review

For peer review only

Abstract

Background:

National and international asthma guidelines recommend adjusting asthma treatment based on levels of control, yet no guidance is given regarding the stepping-down of montelukast in children and young people (CYP).

Objective:

To systematically review evidence regarding deprescribing montelukast in CYP with established asthma.

Design:

Systematic review: PROSPERO ID: CRD42020213971

Data Sources:

Embase, Medline, PubMed and CINAHL were searched up to October 2020.

Study Selection:

Eligible studies contained patients aged 0-18 years with a diagnosis of asthma, who had been administering montelukast before it was withdrawn. All reasons for withdrawal were included.

Results:

The search identified 197 papers. After deduplication, 5 were included (3 randomised control studies and 2 cohort studies). 4 studies observed the impact of montelukast withdrawal for 2 weeks, and 1 study for 8 weeks. Impact of withdrawal was measured in the studies using a combination of lung function tests (e.g. FEV1, FeNO), asthma score systems and exercise challenges. 8 of the 17 the Core Outcome Set for Clinical Trials in Childhood Asthma were measured in at least 1 of the 5 studies, with all 5 studies measuring lung function. No significant differences across comparator points were found between the test and control groups across all studies. Significant differences between the comparator point and posttreatment values were found in 6 different measures across 3 studies; FeNO and eNO significantly increased whereas FEV1, asthma score, fall in FEV1 and recovery time significantly decreased.

Conclusion:

Definitive studies determining clinical stability, and impact of deprescribing montelukast in CYP are imperative to improve the safety of asthma treatment in CYP.

Strengths and limitations of this study

Strengths

For peer review only - http://bmjopen.bmj.com/site/about/guidelines.xhtml

- With montelukast commonly used globally, this review did not limit publication eligibility based on language.
- This systematic review was conducted in accordance with PRISMA guidelines and therefore viewed highly by healthcare professionals (audience).

Limitations

- Some papers had to be excluded due to uncertainties regarding patients' formal asthma diagnosis.

Introduction:

Asthma is a disease of lung inflammation and small airway constriction, and affects more than 338 million people globally [1]. It is the most common chronic disease in children [2]. Asthma can be of variable severity, with symptoms induced by a range of factors such as exercise, viruses or pollen [3]. As a result, the dose and class of asthma medication hugely varies between individuals in order to ensure an effective, personalised treatment plan. This rationale is encouraged by national and international guidelines which aid clinicians in their decision-making [4-8]. However, ambiguities in current guidelines prevent high quality or safe asthma management.

One drug included in most guidelines is montelukast, the 16th most prescribed medication globally in 2020 [9]. Although sometimes prescribed as a first-line treatment, montelukast, a leukotriene receptor antagonist, is commonly prescribed as an additional therapy for patients whose asthma is not controlled by inhaled corticosteroids; its use therefore depends on the needs of a patient at a particular time [7 10]. While the addition and stepping down of treatment is encouraged by asthma guidelines, the process of deprescribing montelukast is not clearly described [4-8]. There is clarity about when montelukast is ineffective, where guidelines state that montelukast treatment should be stopped after an initial trial period [5-7]. However, no montelukast-specific guidance is given for the deprescribing of this drug following the achievement of "good asthma control"- the definition of which is poorly defined [4]. The lack of data regarding the deprescribing of montelukast is not only evident in asthma guidelines but has been noted in literature too [11].

This systematic review aims to collate the current knowledge base around the deprescribing of montelukast in CYP with asthma. The primary aim was to identify the impact of montelukast withdrawal on a patient's asthma systems and control using the Core Outcome Set for Clinical Trials in Childhood Asthma (COS) [12]. The longevity in which the impact of montelukast withdrawal was examined for in the literature was also reviewed.

Methods:

The protocol for this review was registered in the International Prospective Register of Systematic Reviews (PROSPERO 2020: CRD42020213971) at the start of the study (September 2020). Our systematic review is reported in line with the Preferred Reporting Items for Systematic Reviews (PRISMA) guidelines.

Eligibility Criteria

Eligible studies contained patients aged 0-18 years with a diagnosis of asthma and had been administering montelukast before it was withdrawn. Studies which contained both adult (18+ years) and paediatric data were included if the relevant data (information regarding the deprescribing on

For peer review only - http://bmjopen.bmj.com/site/about/guidelines.xhtml

montelukast) were recorded separately from the adult data. Human studies in any language and with any publication date were included. All primary research study designs including case reports, randomised control trials (RCT) and cohort studies were eligible. No narrative reviews or editorials were included. The primary aim was to identify the impact of montelukast withdrawal on a patient's asthma systems and control using the Core Outcome Set for Clinical Trials in Childhood Asthma (COS) [12]. The secondary aim was to review the time over which the withdrawal of montelukast in CYP was measured.

Search Strategy and Study Selection

In September 2020, we searched Medline, PubMed, EMBASE and CINAHL using a combination MeSH and free text subject headings to include the research question (see Table S1 - Supplementary Data Section- for complete search strategy). The primary author (ED) screened the titles and abstracts of all identified studies before comparing the full texts of the remaining studies with the eligibility criteria. This process was repeated independently by the second author (CK) in October 2020. Subsequently the authors finalised the eligible studies; a separate author (DH) independently resolved disagreements between the authors at the full text stage. Reference lists of eligible papers were manually screened for additional papers.

Patient and Public Involvement

No patient involved.

Quality assessment

RTCs were appraised using the Cochrane Collaboration's Risk of Bias Tool [13] and cohort studies using The Newcastle-Ottawa Quality Assessment Form [14] (Tables S2-S3- Supplementary Data Section). This was done by two authors (ED and DH).

Data extraction and synthesis

Data were extracted by one author (ED). Uncertainties encountered during data extraction were independently reviewed by a second author (DH). From each study we detailed characteristics identified in the COS which included the impact of montelukast withdrawal on asthma exacerbations, lung function and quality of life [12]. Results were additionally categorised into short-term (0-6 weeks), medium term (6 weeks to 6 months), and long-term effects (>6 months).

Statistical methods

Results are collated and reported descriptively. Meta-analysis was not appropriate.

Results

After duplicates were removed, the search identified 197 papers. We excluded 182 papers based upon the title and abstract and a further 10 following full-text screening. Five papers met the eligibility criteria (Fig. 1). The eligible papers comprised three RCT and two cohort studies. In total 155 patients were included, of which 107 were administered with and withdrawn from montelukast. Further characteristics of the studies are presented in Table 1.

For peer review only - http://bmjopen.bmj.com/site/about/guidelines.xhtml

For peer review only

For peer review only - http://bmjopen.bmj.com/site/about/guidelines.xhtml

Table 1: Eligible studies| Details of the studies which met the eligibility criteria.

Study							Patients				
**Name of Study**	**Authors**	**Date**	**Study Type**	**Duration of Study (weeks)**	**Study Design**	**Patients Who Completed the Study**	**Patients Given Montelukast (n)**	**Age of Patients (years)**	**Asthma Status Inclusion Criteria**	**Dose of Montelukast (mg)**	**Other Drugs Permited During Study**
Airway mechanics after withdrawal of a leukotriene receptor antagonist in children with mild persistent asthma: Double-blind, randomized, cross-over study	Kim J.H.; Lee S.; Han M.Y. *et al.*	2020	Randomised, double-blind, placebo controlled, cross-over study	5	Placebo or montelukast for 2 weeks, washout for 1 week and swap treatment for 2 weeks.	28	28	7.1 (mean)	1) On Global Initiative for Asthma guidelines step-2 treatment with Montelukast for 1 month or longer.<br>2) Absence of asthma exacerbations or respiratory systems at least 1 month before study onset.<br>3) No use of systemic corticosteroids during the previous 4 weeks.	4 or 5	Short-acting bronchodilator
Exhaled nitric oxide before and after montelukast sodium therapy in school-age children with chronic asthma: A preliminary study	Bratton D.L.; Lanz M.J.; Miyazawa N. *et al.*	1999	Cohort study	8	2 weeks run-in, treatment for 4 weeks, 2-week washout. Controls (matched by age and gender, no history of asthma) were given no placebo.	24	12	6- 11	1) Clinical history of mild to moderate stable asthma.<br>2) Presence of symptoms requiring beta-agonist therapy on at least 7 of the 14 days ruing the run-in period with evidence of airway reversibility (> 12% improvement in FEV1).<br>3) Judged to be in good health on the basis of history and physical examination.	5	Constant dose of nasal corticosteroids

For peer review only - http://bmjopen.bmj.com/site/about/guidelines.xhtml

Effects of montelukast treatment and withdrawal on fractional exhaled nitric oxide and lung function in children with asthma	Montuschi P.; Mondino C.; Koch P. *et al.*	2007	Randomised, double-blind, placebo controlled, parallel group study	7	1 week run-in, treatment or placebo for 4 weeks, 2 weeks washout.	26	14	10.5 (mean, placebo)<br><br>10.8 (mean, treatment)	1) Have mild persistent asthma defined by the National Heart, Lung and Blood Institute of the National Institutes of Health.<br>2) Have symptoms more often than twice a week but less often than once a day.<br>3) FEV1 > 80% of predicted value and reversibility of > 12 % to salbutamol or a positive provocation test result with methacholine or exercise.<br>4) No leukotriene receptor antagonist in the previous 4 weeks.<br>5) No glucocorticoids for > 4 weeks in previous year.	5	Short-acting bronchodilator
Effects of montelukast on symptoms and eNO in children with mild to moderate asthma	Lee M.Y.; Lai Y.S.; Yang K.D. *et al.*	2005	Cohort study	6	2 weeks run-in, treatment for 2 weeks, 2 weeks washout.	13	13	6- 14	1) Have mild to moderate persistent asthma as defined by the National Asthma Education and Prevention Program, Expert Panel Report II, Guidelines for the Diagnosis and Management of Asthma.<br>2) No corticosteroids or leukotriene D4 receptor antagonist treatment in the previous 4 weeks.	5	Short-acting bronchodilator
Prolonged effect of montelukast in asthmatic children with exercise-induced bronchoconstriction	Kim J.H.; Lee S.Y.; Kim H.B. *et al.*	2005	Randomised, parallel-group study	16	treatment or placebo for 8 weeks (db), 8 weeks washout (sb).	64	Period 1: 40 Period 2: 12	8- 14 (mean, 10.3 treatment)<br><br>(10.4 mean, placebo)	1) Have mild asthma defined by the American Thoracic Society.<br>2) History of exercise-induced bronchoconstriction.<br>3) FEV1 of > 80% predicted and a decrease of >15 % after standardised exercise challenge at screening.<br>4) No corticosteroids, long-acting beta-agonists or leukotriene receptor antagonists.	5	Short-acting bronchodilator

For peer review only

Risk of Bias of Included Studies

Tables S2-S3 show the results of the risk of bias assessments for the five papers examined. Both cohort studies show an overall low risk of bias, but one (Lee *et al.,* 2005) demonstrated some uncertainly due to the selection of the non-exposed cohort (no control group) [15 16]. The RCTs were judged to have low risk [17-19].

Montelukast Withdrawal: Core Outcomes Set

Across the five eligible studies, the impact of withdrawing montelukast from children with asthma was only measured against eight of the 17 COS (Table 2). A total of 18 outcomes, measured using 13 unique measures, were used to quantify change in individuals before and after montelukast was withdrawn.

*Table 2: Presence of Core Outcome Set in Studies| *Factor included in asthma severity score. Full details of all asthma severity scores were not detailed in the studies.*

Domains of COS	Core Outcome Set for School-Aged Children with Asthma	Kim *et al.*, 2020	Bratton *et al.*, 1999	Montuschi *et al.*, 2007	Lee *et al.*, 2005	Kim *et al.*, 2005
Symptom Control	Daytime Symptoms	+*	-	-	+*	+*
	Death	-	-	-	-	-
	Exacerbations	+*	-	-	+*	-
	Lung Function Tests	+	+	+	+	+
	Nocturnal Symptoms	+*	-	-	+*	+*
	Parent/ Child Global Assessment of Control	+	-	-	-	-
	Use of Reliever Inhaler	-	+	-	+	-
General Health Impact	Growth	-	-	-	-	-
	Health-Related Problems when Older	-	-	-	-	-
	Long-Term Adverse Effect	-	-	-	-	-
	Short-Term Adverse Effect	-	-	-	+	+
Healthcare Required	GP/ A + E Attendance	-	-	-	-	-
	Hospital Admission	-	-	-	-	-
Life Factors	Activity or Exercise	+*	-	-	-	+
	Normal Activities	-	-	-	-	-
	Quality of Life	-	-	-	-	-
	School Attendance	-	-	-	-	-

Four studies measured symptoms at the point the patient had completed montelukast treatment (therefore exposed to montelukast therapy), whereas one (Kim *et al.,* 2020) compared pre-montelukast symptoms with those after exposure following a subsequent period off treatment. No significant difference was found between treatment groups between the comparator point and posttreatment (washout) periods for any of the 18 outcomes measured (Table 3). However there were significant differences between the comparator points within the test groups (Table 4).

For peer review only - http://bmjopen.bmj.com/site/about/guidelines.xhtml

*Table 3: Deprescribing montelukast| The recorded outcomes of deprescribing montelukast in children with established asthma. *Based upon the mean age of the participants, we have assumed the C-ACT used was for children aged 4 to 11 years.*

**Study**	**Length of time prescribed Montelukast**	**Final treatment to post-treatment phase**	**Measure**	**Timepoint of comparator measurement**	**Difference across comparator points between montelukast and control groups**
Kim et al., 2020	2 weeks	2 weeks	FEV1	Baseline	No significant difference was found between treatment groups between baseline and posttreatment
			FEV1/FVC		No significant difference was found between treatment groups between baseline and posttreatment
			IOS		No significant difference was found between treatment groups between baseline and posttreatment
			FeNO		No significant difference was found between treatment groups between baseline and posttreatment
			C-ACT*		No significant difference was found between treatment groups between baseline and posttreatment
			FEV1/BDR		No significant difference was found between treatment groups between baseline and posttreatment
Bratton *et al.*, 1999	4 weeks	2 weeks	eNO	End of treatment	No comparison was made between the treatment group and the control group during the washout phase.
Montuschi *et al.*, 2007	4 weeks	2 weeks	FeNO	End of treatment	No significant difference between the treatment group and the control group during the washout phase
			FEV1		No significant difference between the treatment group and the control group during the washout phase
			FEV1/FVC %		No significant difference between the treatment group and the control group during the washout phase
			FEF (25%-75%)		No significant difference between the treatment group and the control group during the washout phase
Lee *et al.*, 2005	2 weeks	2 weeks	eNO	End of treatment	No control or placebo used
			PEFR		
			Asthma Score		
			Asthma Attack		
Kim *et al.*, 2005	8 weeks	8 weeks	Asthma Score	End of treatment	No comparison was made between the washout group (placebo) and the treatment group (deprescribing data)
			Maximum % fall in FEV1		
			Time to Recovery		

For peer review only

Table 4: Significant differences in measures between the comparator point used in each study (Table 3) and following montelukast withdrawal.

Study	Measure with Significant Change Across Studies	Significant Change Between Comparator Point and Post Treatment (Washout) of Test Group	Value at Comparator Point	Value Posttreatment
Montuschi *et al.*, 2007	FeNO	Significantly increased (p = 0.023)	37.9 ppb (25.6-62.5) (median (interquartile range))	52.2 ppb (36.0-72.9) (median (interquartile range))
	FEV1	Significantly decreased (p = 0.011)	2.48 L ± 0.22 (mean ± SEM)	2.33 ± 0.19 (mean ± SEM)
Lee *et al.*, 2005	eNO	Significantly increased (p = 0.011)	13.5 ppb ± 7.60 (mean ± SD)	29.2 ppb ± 16.1 (mean ± SD)
Kim *et al.*, 2005	Asthma Score	Significantly decreased (p <0.050)	17.8 ± 6.8 (mean ± SD)	17.7 ± 6.78 (mean ± SD)
	Maximum % fall in FEV1	Significantly decreased (p <0.050)	27.6% ± 14.4 (mean ± SD)	26.7 % ± 19.4 (mean ± SD)
	Time to Recovery	Significantly decreased (p <0.050)	25.3 mins ± 23.3 (mean ± SD)	27.7 mins ± 26.5 (mean ± SD)

For peer review only

Exacerbation

Kim, *et al* [18] used the C-ACT questionnaire with the purpose to assess asthma control in both the placebo and test group [20]. No significant difference was found between changes in C-ACT score between treatment groups or the comparator points. Lee, *et al* [16] also used an asthma scoring system which monitored factors including asthma exacerbations. Asthma scores did not significantly change during the study. Full details of this asthma scoring system were not able to be identified. In addition, Lee, *et al* [16] recorded that two of the 13 patients experienced mild asthma attacks during the two-week washout period post montelukast administration, demonstrating a worsening of asthma control (Table 3).

Daytime and Nocturnal Symptoms

The C-ACT questionnaire used by Kim, *et al* [18] included an assessment of daytime and nocturnal symptoms [20]. No significant difference was found between changes in C-ACT score between the placebo and test groups or the comparator points [18]. The asthma scoring systems used by Lee, *et al* [16] also described monitoring factors including daytime and nocturnal symptoms. The asthma score did not significantly change during the study. However, there was a significant decrease (p <0.050) in the asthma score (improved symptom control) used by Kim, *et al* [17] which accounted for daytime and night-time asthma symptoms (Table 4). Full details of this asthma scoring system were not able to be identified [17].

For peer review only - http://bmjopen.bmj.com/site/about/guidelines.xhtml

Activity or exercise

The C-ACT questionnaire used by Kim, *et al* [18] included an assessment of exercise [20]. No significant difference was found between changes in C-ACT score between the placebo and test groups or between the comparator points. However, in the study conducted by Kim, *et al* [17] the recovery time for a patient's FEV1 to return within 10% of their pre-exercise baseline following a standardised exercise challenge significantly decreased (p <0.050) between the end of montelukast administration and the end of the washout period, demonstrating an improvement in asthma control (Table 4).

Short-term adverse effects

Reports of a mild headache subsisting spontaneously two days after montelukast withdrawal were recorded by Lee, *et al* [16].

Lung Function Tests

All five studies undertook lung function tests to assess the impact of montelukast withdrawal on lung function.

The change in FEV1 between the comparator point and the end of the washout phase was measured in two of the five studies (Table 3) [18 19]. Montuschi *et al.*[19] recorded a significant decrease (p=0.011) in FEV1 demonstrating a reduced pulmonary function following montelukast withdrawal (Table 4) whereas Kim *et al.* [18] recorded no significant change. However, Kim *et al.* [17] recorded a significant decrease (p<0.050, Table 4) in the maximum % fall in FEV1 in the period following montelukast withdrawal (Table 3), demonstrating an increase in pulmonary function.

Unlike Kim *et al.* [18], Montuschi *et al.*[19] recorded a significant increase (p=0.023) in FeNO following montelukast withdrawal, demonstrating a reduced pulmonary function (Table 3 and Table 4). Changes in eNO recorded by Lee *et al.* also demonstrate a reduced pulmonary function (significant increase, p=0.011) during the washout period (Table 4) yet Bratton *et al.* recorded a non-significant change for the same measure (Table 3). Measures of FEV1/FVC, FEF (25%-75%), FEV1/BDR, PEFR and IOS were universally insignificant across the five studies (Table 3).

Timeframe

Four studies [15 16 18 19] observed the impact of montelukast withdrawal for two weeks (short-term) and one study [17] for eight weeks (medium-term). No study examined the long-term effects (>6 months) of montelukast withdrawal.

Discussion

Deprescribing or stepping down therapy is always a time of risk for the patient. However, a change in therapy is not uncommon amongst asthma patients as their therapeutic regime is governed the control of their asthma symptoms [4 7]. Factors which may induce a change in asthma symptom severity include changes in weather and pollen levels, or the improvement of asthma control over time [21]. As montelukast is recommended as an "add-on" therapy in CYP with more difficult to control asthma, the addition or removal of this drug from a patient's drug regime is not uncommon; it is recommended by international and national guidelines that CYP with asthma should be treated at the lowest "step" of treatment which manages their symptoms [4 7 22]. The interest in stepping down montelukast has heightened since the FDA applied a boxed warning to montelukast in March 2020 due to the increasing number of neuropsychiatric events reported [23 24]. Since then, further

For peer review only - http://bmjopen.bmj.com/site/about/guidelines.xhtml

concerns regarding the adverse drug reactions attributed to montelukast in CYP with asthma are being outlined in an ongoing PROSPERO systematic review (ID: CRD42020209627).

Regardless that deprescribing montelukast should be commonplace, this review highlights the limited number of studies which have examined the stepping down of montelukast in CYP with diagnosed asthma. Additionally, the information contained in the studies demonstrates the knowledge deficit in this area of the medicinal management of childhood asthma. Firstly the five eligible papers primarily examine lung function as a proxy for asthma. Measuring lung function is a commonplace technique used to understand and aid the diagnosis of asthma. However, for most methods of measuring lung function, there is very little information about how the measure relates to asthma management in children in RCT or large longitudinal studies [25]. For example, in 2017 Murray *et al.* concluded that the diagnostic algorithm, which uses data from FEV1, FeNO and FVC measurements, was accurately poor [26]. Therefore, the use of lung function measures, of which make up 14 of the 18 measures undertaken across the eligible studies, are not reliable in understanding the impact of montelukast withdrawal on the asthma control of children.

Additionally, this review identifies the lack of relevant core outcomes examined within the literature, with the studies found examining only a maximum of six of the 17 outcomes (Table 2) [12]. As a result, the outlined standard of holistic and physiological understanding monitoring the stepping down of montelukast in CYP has not been met, compromising the safety of CYP and the standard of care given. With asthma being the most common, non-communicable disease of childhood, it is imperative that this COS for deprescribing montelukast is evaluated as a matter of urgency [27].

Lastly, the studies examine the effect of the withdrawal of montelukast over a short time frame relative to the expected length of treatment. With four of the five studies only observing the patients for two week post final montelukast administration, only some aspects of the immediate washout phase are known. As a result, the only knowledge found in literature regarding the effects of deprescribing montelukast after two weeks come from a single paper [17]. Thus, further studies examining the deprescribing of montelukast in CYP with asthma are necessary to evaluate whether the findings of these studies are maintained over a longer posttreatment period.

Strengths and Limitations of this Study

The small number of papers, and therefore the small volume of data identified in this review amplifies the need for further studies examining the deprescribing of montelukast in CYP with established asthma. As none of the five studies came close to meeting the COS for trials of childhood asthma, this review also highlights aspects of asthma management that are unknown. Therefore, not only does this review illuminate the need for additional studies to better understand this asthmatic population but it identifies specific areas in which future studies must investigate in order for research-led medicine to be practised.

However, a limitation of this review is the unknown contents of the two asthma scoring systems used by Lee *et al.* and Kim *et al.* [16 17]. Although some of the factors included in these scores are listed in their publications (e.g. night-time symptoms) and therefore included in Table 2, the extent of the scores could not be examined as part of this review. As a result, it is possible that certain factors outlined in by the COS for trials of childhood asthma were examined as part of the scoring systems used. Additionally, it is possible that individual factors examined in the scoring systems did significantly change between the comparator point and the washout period, but the overall asthma score did not and therefore the change in the factor was masked. However, it can be argued that if

For peer review only - http://bmjopen.bmj.com/site/about/guidelines.xhtml

additional COS factors were examined as part of the scoring systems, their absence from the main text of the paper suggests that their examination by the authors was insignificant.

Conclusion

To conclude, only limited, contradictory and short-term effects of deprescribing montelukast in CYP with established asthma are present in literature. Definitive studies determining clinical stability, and impact of deprescribing montelukast in CYO are imperative in order for guidelines to fully reflect the safety and holistic impact of stepping down this treatment in children.

Funding

This study did not receive specific funding.

Conflict of Interests

No conflicts of interest were declared.

Acknowledgements

This is a summary of independent research carried out at the National Institute for Health Research (NIHR) Alder Hey Clinical Research Facility. The views expressed are those of the author(s) and not necessarily those of the NHS, the NIHR or the Department of Health.

References

1. Ferrante G, La Grutta S. The Burden of Pediatric Asthma. *Frontiers in Pediatrics* 2018;6(186) doi: 10.3389/fped.2018.00186
2. World Health Organisation. Asthma https://www.who.int/news-room/q-a-detail/asthma: World Health Organisation,; 2020 [updated 15/05/2020. Available from: https://www.who.int/news-room/q-a-detail/asthma accessed 09/09/2020 2020.
3. Colice GL. Categorizing asthma severity: an overview of national guidelines. *Clin Med Res* 2004;2(3):155-63. doi: 10.3121/cmr.2.3.155
4. Global Initiative for Athma. Global Strategy for Asthma Management and Prevention, 2020.
5. National Asthma Council Australia. Australian Asthma Handbook, Quick Reserence Guide, 2014.
6. National Institute for Health and Care Excellence. Asthma: diagnosis, monitoring and chronic asthma management 2017 [Available from: https://www.nice.org.uk/guidance/ng80/resources/asthma-diagnosis-monitoring-and-chronic-asthma-management-pdf-1837687975621 accessed 03/02/21 2021.
7. British Thoracic Society and The Scottish Intercollegiate Guidelines Network. SIGN158 British Guidelines on the Management of Asthma [Clinical Guidelines]. https://www.brit-thoracic.org.uk/quality-improvement/guidelines/asthma/2019 [updated July 2020; cited 2020 09/09/2020]. Available from: https://www.brit-thoracic.org.uk/quality-improvement/guidelines/asthma/ accessed 09/09/2020 2020.
8. Chung KF, Wenzel SE, Brozek JL, et al. International ERS/ATS guidelines on definition, evaluation and treatment of severe asthma. *European Respiratory Journal* 2014;43(2):343-73. doi: 10.1183/09031936.00202013
9. OpenPrescribing. Montelukast (0303020G0) https://openprescribing.net/chemical/0303020G0/2020 [updated 01/06/2020. Available from: https://openprescribing.net/chemical/0303020G0/ accessed 14/09/2020 2020.

For peer review only - http://bmjopen.bmj.com/site/about/guidelines.xhtml

For peer review only

10. Tamada T, Ichinose M. Leukotriene Receptor Antagonists and Antiallergy Drugs. *Handb Exp Pharmacol* 2017;237:153-69. doi: 10.1007/164_2016_72 [published Online First: 2016/11/09]
11. King C, Dixon EG, Hawcutt DB. Stepping down asthma treatment in children. *Pediatric Pulmonology* 2021 doi: 10.1002/ppul.25410
12. Sinha IP, Gallagher R, Williamson PR, et al. Development of a core outcome set for clinical trials in childhood asthma: a survey of clinicians, parents, and young people. *Trials* 2012;13(1):103. doi: 10.1186/1745-6215-13-103
13. Higgins JPT, Altman DG, Gøtzsche PC, et al. The Cochrane Collaboration's tool for assessing risk of bias in randomised trials. *BMJ* 2011;343:d5928. doi: 10.1136/bmj.d5928
14. Wells G, Shea B, O'Connell D, et al. The Newcastle-Ottawa Scale (NOS) for assessing the quality of nonrandomised studies in meta-analyses http://www.ohri.ca/programs/clinical_epidemiology/oxford.asp: The Ottawa Hospital Research Institute; 2019 [Available from: http://www.ohri.ca/programs/clinical_epidemiology/oxford.asp accessed 08/12/2020 2020.
15. Bratton DL, Lanz MJ, Miyazawa N, et al. Exhaled nitric oxide before and after montelukast sodium therapy in school-age children with chronic asthma: a preliminary study. *Pediatr Pulmonol* 1999;28(6):402-7. doi: 10.1002/(sici)1099-0496(199912)28:6<402::aid-ppul3>3.0.co;2-v [published Online First: 1999/12/10]
16. Lee M-Y, Lai Y-S, Yang KD, et al. Effects of montelukast on symptoms and eNO in children with mild to moderate asthma. *Pediatrics International* 2005;47(6):622-26. doi: 10.1111/j.1442-200x.2005.02142.x
17. Kim J-H, Lee S-Y, Kim H-B, et al. Prolonged effect of montelukast in asthmatic children with exercise-induced bronchoconstriction. *Pediatric Pulmonology* 2005;39(2):162-66. doi: 10.1002/ppul.20156
18. Kim JH, Lee S, Shin YH, et al. Airway mechanics after withdrawal of a leukotriene receptor antagonist in children with mild persistent asthma: Double-blind, randomized, cross-over study. *Pediatric Pulmonology* 2020;55(12):3279-86. doi: https://doi.org/10.1002/ppul.25085
19. Montuschi P, Mondino C, Koch P, et al. Effects of montelukast treatment and withdrawal on fractional exhaled nitric oxide and lung function in children with asthma. *Chest* 2007;132(6):1876-81.
20. Nathan RA, Sorkness CA, Kosinski M, et al. Childhood Asthma Control Test for Children 4 to 11 Years. 2004 [Available from: https://www.greenhillspeds.com/wp-content/uploads/2015/12/Asthma-Control-Test-4-to-11-years.pdf.
21. Asthma UK. What is asthma? 2019 [updated 01/2019; cited 2021 16/02]. Available from: https://www.asthma.org.uk/advice/understanding-asthma/what-is-asthma/.
22. Electronic Medicines Compendium. Montelukast 5mg Chewable Tablets https://www.medicines.org.uk/emc/product/6097/smpc#gref2011 [updated 23/05/2011. Available from: https://www.medicines.org.uk/emc/product/6097/smpc#gref accessed 09/12/20 2020.
23. U.S. Food and Drug Administration. FDA Requires Stronger Warning About Risk of Neuropsychiatric Events Associated with Asthma and Allergy Medication Singulair and Generic Montelukast. FDA Requires Stronger Warning About Risk of Neuropsychiatric Events Associated with Asthma and Allergy Medication Singulair and Generic Montelukast, 2020.
24. E.J. MUNDELL. Allergy Med Singulair to Get 'Black Box' Warning Over Psych Side Effects: FDA. *US News* 2020.
25. Moeller A, Carlsen K-H, Sly PD, et al. Monitoring asthma in childhood: lung function, bronchial responsiveness and inflammation. *European Respiratory Review* 2015;24(136):204-15. doi: 10.1183/16000617.00003914
26. Murray C, Foden P, Lowe L, et al. Diagnosis of asthma in symptomatic children based on measures of lung function: an analysis of data from a population-based birth cohort study.

For peer review only

For peer review only - http://bmjopen.bmj.com/site/about/guidelines.xhtml

The Lancet Child & Adolescent Health 2017;1(2):114-23. doi: 10.1016/s2352-4642(17)30008-1

27. Asthma UK. Asthma facts and statistics 2020 [cited 2021 16/02]. Available from: https://www.asthma.org.uk/about/media/facts-and-statistics/.

For peer review only

For peer review only - http://bmjopen.bmj.com/site/about/guidelines.xhtml

For peer review only

For peer review only - http://bmjopen.bmj.com/site/about/guidelines.xhtml

For peer review only

For peer review only - http://bmjopen.bmj.com/site/about/guidelines.xhtml

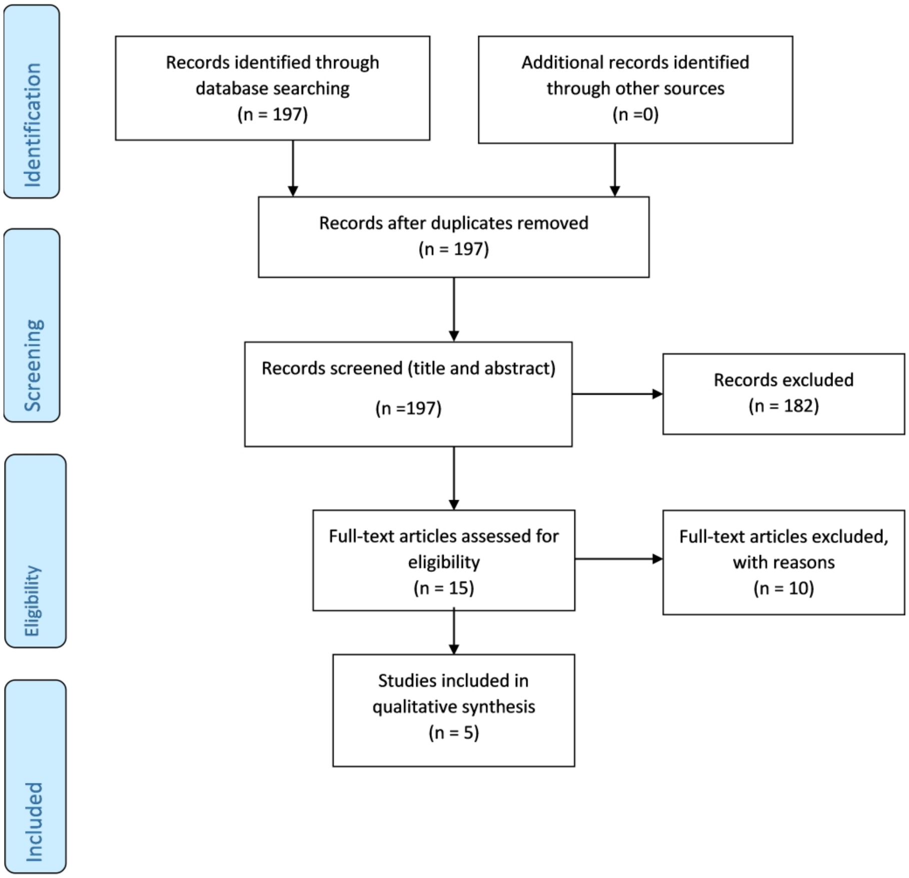

Figure 1: Collection of Sources

1233x1175mm (96 x 96 DPI)

Deprescribing Montelukast in Children with Asthma: A Systematic Review (SUPPLEMENTARY DATA SECTION)

Eleanor Dixon[1,2], Charlotte King[3], Andrew Lilley[4], Ian Sinha[5], Daniel B Hawcutt[2,4]

1: Department of Pharmacology and Therapeutics, University of Liverpool, UK

2: Department of Women's and Children's Health, University of Liverpool, UK

3: Liverpool University Hospital Trust, Liverpool, UK

4: NIHR Alder Hey Clinical Research Facility, Alder Hey Children's Hospital, Liverpool, UK

5: Department of Respiratory Medicine, Alder Hey Children's Hospital, Liverpool, UK

Tables: 3

Keywords: Montelukast, Adverse Drug Reactions, Asthma, Paediatric, Systematic Review

For peer review only

Methods

Table S1: Search Strategy

Strategy 918451 #	Database	Search term	Results
1	EMBASE	CHILD/ OR INFANT/ OR BABY/ OR NEWBORN/	2417196
2	EMBASE	JUVENILE/ OR CHILD/ OR BOY/ OR GIRL/ OR INFANT/ OR TODDLER/ OR "SCHOOL CHILD"/ OR "PRESCHOOL CHILD"/	2301714
3	EMBASE	JUVENILE/ OR ADOLESCENT/	1563364
4	EMBASE	PEDIATRICS/	78300
5	EMBASE	((Paediatric* OR Infan* OR Newborn* OR Bab* OR Neonat* OR Preterm* OR Prematur* OR postmatur* OR Child* OR School* OR "Nursery school*" OR Kindergar* OR "Primary school*" OR "Secondary school*" OR "Elementary school*" OR "High school*" OR Highschool* OR Preschool* OR Kid* OR Toddler* OR Adoles* OR Teen* OR Boy* OR Girl* Minor* OR Pubert* OR Pubescen* OR Prepubescen*) NOT REVIEW*).ti,ab	3217377
6	EMBASE	"MINOR (PERSON)"/	653
7	EMBASE	ADOLESCENCE/ OR PUBERTY/ OR PREPUBERTY/	70704
8	EMBASE	(1 OR 2 OR 3 OR 4 OR 5 OR 6 OR 7)	4839396
9	EMBASE	8 [Humans]	4025357
10	EMBASE	ASTHMA/ OR "ASTHMA BRONCHIALE"/	231772
11	EMBASE	((Asthma* OR Wheez* OR "Bronchial asthma*" OR "stable asthma") NOT REVIEW).ti,ab	216313
12	EMBASE	(10 OR 11)	284696
13	EMBASE	12 [Humans]	238534
14	EMBASE	DEPRESCRIPTION/ OR DEPRESCRIPTIONS/	501
15	EMBASE	"TREATMENT WITHDRAWAL"/ OR "DRUG WITHDRAWAL"/	215067
16	EMBASE	((deprescrib* OR stop* OR withdraw* OR ceas* OR discontinu* OR de-prescrib*) NOT REVIEW).ti,ab	536525
17	EMBASE	(14 OR 15 OR 16)	680842
18	EMBASE	17 [Humans]	519666
19	EMBASE	MONTELUKAST/	9658
20	EMBASE	((montelukast OR singular) NOT REVIEW).ti,ab	15377
21	EMBASE	(19 OR 20)	21572
22	EMBASE	21 [Humans]	14849
23	EMBASE	(8 AND 12 AND 17 AND 21)	194
24	EMBASE	23 [Humans]	191
25	Medline	CHILD/ OR "CHILD, PRESCHOOL"/	1916435
26	Medline	ADOLESCENT/ OR MINORS/	2036089
27	Medline	INFANT/ OR "INFANT, NEWBORN"/	1139739

For peer review only

28	Medline	((Paediatric* OR Infan* OR Newborn* OR Bab* OR Neonat* OR Preterm* OR Prematur* OR postmatur* OR Child* OR School* OR "Nursery school*" OR Kindergar* OR "Primary school*" OR "Secondary school*" OR "Elementary school*" OR "High school*" OR Highschool* OR Preschool* OR Kid* OR Toddler* OR Adoles* OR Teen* OR Boy* OR Girl* Minor* OR Pubert* OR Pubescen* OR Prepubescen*) NOT REVIEW*).ti,ab	2716250
29	Medline	PUBERTY/	13325
30	Medline	(25 OR 26 OR 27 OR 28 OR 29)	4733651
31	Medline	ASTHMA/	125895
32	Medline	((Asthma* OR Wheez* OR "Bronchial asthma*" OR "stable asthma") NOT REVIEW).ti,ab	155279
33	Medline	(31 OR 32)	177833
34	Medline	DEPRESCRIPTIONS/	466
35	Medline	((deprescrib* OR stop* OR withdraw* OR ceas* OR discontinu* OR de-prescrib*) NOT REVIEW).ti,ab	369364
36	Medline	(34 OR 35)	369497
37	Medline	((montelukast OR singular) NOT REVIEW).ti,ab	13162
38	Medline	(30 AND 33 AND 36 AND 37)	54
39	Medline	38 [Humans]	50
40	PubMed	((Paediatric* OR Infan* OR Newborn* OR Bab* OR Neonat* OR Preterm* OR Prematur* OR postmatur* OR Child* OR School* OR "Nursery school*" OR Kindergar* OR "Primary school*" OR "Secondary school*" OR "Elementary school*" OR "High school*" OR Highschool* OR Preschool* OR Kid* OR Toddler* OR Adoles* OR Teen* OR Boy* OR Girl* Minor* OR Pubert* OR Pubescen* OR Prepubescen*) NOT REVIEW*).ti,ab	143769
41	PubMed	((Asthma* OR Wheez* OR "Bronchial asthma*" OR "stable asthma") NOT REVIEW).ti,ab	161822
42	PubMed	((deprescrib* OR stop* OR withdraw* OR ceas* OR discontinu* OR de-prescrib*) NOT REVIEW).ti,ab	361553
43	PubMed	((montelukast OR singular) NOT REVIEW).ti,ab	13080
44	PubMed	(40 AND 41 AND 42 AND 43)	0
45	CINAHL	CHILD/ OR INFANT/ OR ADOLESCENCE/ OR "MINORS (LEGAL)"/ OR "CHILD, PRESCHOOL"/	892377
46	CINAHL	PEDIATRICS/ OR NEONATOLOGY/	20791
47	CINAHL	PUBERTY/	3060
48	CINAHL	ADOLESCENCE/ OR CHILD/ OR "MINORS (LEGAL)"/	778755
49	CINAHL	((Paediatric* OR Infan* OR Newborn* OR Bab* OR Neonat* OR Preterm* OR Prematur* OR postmatur* OR Child* OR School* OR "Nursery school*" OR Kindergar* OR "Primary school*" OR "Secondary school*" OR "Elementary school*" OR "High school*" OR Highschool* OR Preschool* OR Kid* OR Toddler* OR Adoles* OR Teen* OR Boy* OR Girl* Minor* OR Pubert* OR Pubescen* OR Prepubescen*) NOT REVIEW*).ti,ab	841196
50	CINAHL	(45 OR 46 OR 47 OR 48 OR 49)	1279403
51	CINAHL	ASTHMA/	34317
52	CINAHL	((Asthma* OR Wheez* OR "Bronchial asthma*" OR "stable asthma") NOT REVIEW).ti,ab	36601
53	CINAHL	((deprescrib* OR stop* OR withdraw* OR ceas* OR discontinu* OR de-prescrib*) NOT REVIEW).ti,ab	73381
54	CINAHL	(51 OR 52)	45034
55	CINAHL	MONTELUKAST/	368
56	CINAHL	((montelukast OR singular) NOT REVIEW).ti,ab	1953
57	CINAHL	(55 OR 56)	2122
58	CINAHL	(50 AND 53 AND 54 AND 57)	14

For peer review only

Results

Table S2: Results of assessment of risk bias of randomised control trials| The Cochrane Collaboration's tool was used. Green= low risk of bias, Yellow= unclear risk of bias, Red= high risk of bias.

	**Kim *et al.*, 2020**	**Montuschi *et al.*, 2007**	**Kim *et al.*, 2005**
Random sequence generation (selection bias)	Green	Yellow	Yellow
Allocation concealment (selection bias)	Green	Yellow	Yellow
Binding of participants and personnel (performance bias)	Green	Green	Green
Binding of outcome assessment (detection bias)	Green	Yellow	Green
Incomplete outcome data (attrition Bias)	Green	Green	Green
Selective reporting (reporting bias)	Green	Green	Green
Other bias	Green	Green	Green

Table S3: Results of assessment of risk bias of case-controlled and cohort studies| The Newcastle-Ottawa Quality Assessment Form was used. Green= star (low risk of bias), Yellow= unknown risk of bias, Red= No star (high risk of bias).

	**Lee *et al.*, 2005**	**Bratton *et al.*, 1999**
Representativeness of the exposed cohort (selection bias)	Green	Green
Selection of the non-exposed cohort (selection bias)	Red	Green
Ascertainment of exposure (selection bias)	Green	Green
Outcome of interest stated (selection bias)	Green	Green
Comparability bias	Red	Green
Assessment of outcome (outcome bias)	Green	Green
Appropriate follow-up of outcomes (outcome bias)	Yellow	Yellow
Appropriate follow-up of patients (outcome bias)	Green	Green
**Overall quality**	Green	Green

For peer review only

For peer review only - http://bmjopen.bmj.com/site/about/guidelines.xhtml

PRISMA 2020 Checklist

Section and Topic	Item #	Checklist item	Location where item is reported
**TITLE**			
Title	1	Identify the report as a systematic review.	Page 1
**ABSTRACT**			
Abstract	2	See the PRISMA 2020 for Abstracts checklist.	Page 2
**INTRODUCTION**			
Rationale	3	Describe the rationale for the review in the context of existing knowledge.	Page 3
Objectives	4	Provide an explicit statement of the objective(s) or question(s) the review addresses.	Page 3
**METHODS**			
Eligibility criteria	5	Specify the inclusion and exclusion criteria for the review and how studies were grouped for the syntheses.	Page 3
Information sources	6	Specify all databases, registers, websites, organisations, reference lists and other sources searched or consulted to identify studies. Specify the date when each source was last searched or consulted.	Page 3-4 and supplementary
Search strategy	7	Present the full search strategies for all databases, registers and websites, including any filters and limits used.	Supplementary
Selection process	8	Specify the methods used to decide whether a study met the inclusion criteria of the review, including how many reviewers screened each record and each report retrieved, whether they worked independently, and if applicable, details of automation tools used in the process.	Page 4
Data collection process	9	Specify the methods used to collect data from reports, including how many reviewers collected data from each report, whether they worked independently, any processes for obtaining or confirming data from study investigators, and if applicable, details of automation tools used in the process.	Page 4
Data items	10a	List and define all outcomes for which data were sought. Specify whether all results that were compatible with each outcome domain in each study were sought (e.g. for all measures, time points, analyses), and if not, the methods used to decide which results to collect.	Page 4
	10b	List and define all other variables for which data were sought (e.g. participant and intervention characteristics, funding sources). Describe any assumptions made about any missing or unclear information.	Page 4
Study risk of bias assessment	11	Specify the methods used to assess risk of bias in the included studies, including details of the tool(s) used, how many reviewers assessed each study and whether they worked independently, and if applicable, details of automation tools used in the process.	Page 4 and supplementary
Effect measures	12	Specify for each outcome the effect measure(s) (e.g. risk ratio, mean difference) used in the synthesis or presentation of results.	Tables
Synthesis methods	13a	Describe the processes used to decide which studies were eligible for each synthesis (e.g. tabulating the study intervention characteristics and comparing against the planned groups for each synthesis (item #5)).	Page 3-4
	13b	Describe any methods required to prepare the data for presentation or synthesis, such as handling of missing summary statistics, or data conversions.	N/A
	13c	Describe any methods used to tabulate or visually display results of individual studies and syntheses.	Page 3-4
	13d	Describe any methods used to synthesize results and provide a rationale for the choice(s). If meta-analysis was performed, describe the model(s), method(s) to identify the presence and extent of statistical heterogeneity, and software package(s) used.	Page 3-4
	13e	Describe any methods used to explore possible causes of heterogeneity among study results (e.g. subgroup analysis, meta-regression).	N/A
	13f	Describe any sensitivity analyses conducted to assess robustness of the synthesized results.	N/A
Reporting bias assessment	14	Describe any methods used to assess risk of bias due to missing results in a synthesis (arising from reporting biases).	Page 4 and supplementary
Certainty	15	Describe any methods used to assess certainty (or confidence) in the body of evidence for an outcome.	Page 4 and

For peer review only
For peer review only - http://bmjopen.bmj.com/site/about/guidelines.xhtml

PRISMA 2020 Checklist

Section and Topic	Item #	Checklist item	Location where item is reported
assessment			supplementary
**RESULTS**			
Study selection	16a	Describe the results of the search and selection process, from the number of records identified in the search to the number of studies included in the review, ideally using a flow diagram.	Figure 1
	16b	Cite studies that might appear to meet the inclusion criteria, but which were excluded, and explain why they were excluded.	N/A
Study characteristics	17	Cite each included study and present its characteristics.	Page 5-6
Risk of bias in studies	18	Present assessments of risk of bias for each included study.	Supplementary
Results of individual studies	19	For all outcomes, present, for each study: (a) summary statistics for each group (where appropriate) and (b) an effect estimate and its precision (e.g. confidence/credible interval), ideally using structured tables or plots.	N/A
Results of syntheses	20a	For each synthesis, briefly summarise the characteristics and risk of bias among contributing studies.	Page 4
	20b	Present results of all statistical syntheses conducted. If meta-analysis was done, present for each the summary estimate and its precision (e.g. confidence/credible interval) and measures of statistical heterogeneity. If comparing groups, describe the direction of the effect.	Tables
	20c	Present results of all investigations of possible causes of heterogeneity among study results.	Page 4-10
	20d	Present results of all sensitivity analyses conducted to assess the robustness of the synthesized results.	N/A
Reporting biases	21	Present assessments of risk of bias due to missing results (arising from reporting biases) for each synthesis assessed.	N/A
Certainty of evidence	22	Present assessments of certainty (or confidence) in the body of evidence for each outcome assessed.	N/A
**DISCUSSION**			
Discussion	23a	Provide a general interpretation of the results in the context of other evidence.	Page 10-11
	23b	Discuss any limitations of the evidence included in the review.	Page 10-11
	23c	Discuss any limitations of the review processes used.	Page 11
	23d	Discuss implications of the results for practice, policy, and future research.	Page 11
**OTHER INFORMATION**			
Registration and protocol	24a	Provide registration information for the review, including register name and registration number, or state that the review was not registered.	Page 3
	24b	Indicate where the review protocol can be accessed, or state that a protocol was not prepared.	Page 3
	24c	Describe and explain any amendments to information provided at registration or in the protocol.	N/A
Support	25	Describe sources of financial or non-financial support for the review, and the role of the funders or sponsors in the review.	Page 12
Competing interests	26	Declare any competing interests of review authors.	Page 12
Availability of data, code and other materials	27	Report which of the following are publicly available and where they can be found: template data collection forms; data extracted from included studies; data used for all analyses; analytic code; any other materials used in the review.	N/A

For peer review only - http://bmjopen.bmj.com/site/about/guidelines.xhtml

From: Page MJ, McKenzie JE, Bossuyt PM, Boutron I, Hoffmann TC, Mulrow CD, et al. The PRISMA 2020 statement: an updated guideline for reporting systematic reviews. BMJ 2021;372:n71. doi:

For peer review only

PRISMA 2020 Checklist

10.1136/bmj.n71

For more information, visit: http://www.prisma-statement.org/

For peer review only

BMJ Open

Deprescribing Montelukast in Children with Asthma: A Systematic Review

Journal:	*BMJ Open*
Manuscript ID	bmjopen-2021-053112.R1
Article Type:	Original research
Date Submitted by the Author:	12-Oct-2021
Complete List of Authors:	Dixon, Eleanor; University of Liverpool Faculty of Health and Life Sciences, Department of Pharmacology and Therapeutics; University of Liverpool, Department of Women's and Children's Health<br>King, Charlotte; Liverpool University Hospitals NHS Foundation Trust, Royal Liverpool University Hospital<br>Lilley, Andrew; Alder Hey Children's NHS Foundation Trust, Department of Respiratory Medicine<br>Sinha, Ian; Alder Hey Children's NHS Foundation Trust<br>Hawcutt, Daniel; University of Liverpool, Department of Women's and Children's Health; Alder Hey Children's Hospital Clinical Research Facility
**Primary Subject Heading**:	Paediatrics
Secondary Subject Heading:	Pharmacology and therapeutics, Respiratory medicine, Evidence based practice
Keywords:	Asthma < THORACIC MEDICINE, CLINICAL PHARMACOLOGY, PAEDIATRICS

SCHOLARONE™
Manuscripts

I, the Submitting Author has the right to grant and does grant on behalf of all authors of the Work (as defined in the below author licence), an exclusive licence and/or a non-exclusive licence for contributions from authors who are: i) UK Crown employees; ii) where BMJ has agreed a CC-BY licence shall apply, and/or iii) in accordance with the terms applicable for US Federal Government officers or employees acting as part of their official duties; on a worldwide, perpetual, irrevocable, royalty-free basis to BMJ Publishing Group Ltd ("BMJ") its licensees and where the relevant Journal is co-owned by BMJ to the co-owners of the Journal, to publish the Work in this journal and any other BMJ products and to exploit all rights, as set out in our licence.

The Submitting Author accepts and understands that any supply made under these terms is made by BMJ to the Submitting Author unless you are acting as an employee on behalf of your employer or a postgraduate student of an affiliated institution which is paying any applicable article publishing charge ("APC") for Open Access articles. Where the Submitting Author wishes to make the Work available on an Open Access basis (and intends to pay the relevant APC), the terms of reuse of such Open Access shall be governed by a Creative Commons licence – details of these licences and which Creative Commons licence will apply to this Work are set out in our licence referred to above.

Other than as permitted in any relevant BMJ Author's Self Archiving Policies, I confirm this Work has not been accepted for publication elsewhere, is not being considered for publication elsewhere and does not duplicate material already published. I confirm all authors consent to publication of this Work and authorise the granting of this licence.

For peer review only

Deprescribing Montelukast in Children with Asthma: A Systematic Review

For peer review only

Eleanor G. Dixon[1,2], Charlotte King[3], Andrew Lilley[4], Ian Sinha[4], Daniel B Hawcutt[2,5],

1: Department of Pharmacology and Therapeutics, University of Liverpool, UK

2: Department of Women's and Children's Health, University of Liverpool, UK

3: Liverpool University Hospital Trust, Liverpool, UK

4: Department of Respiratory Medicine, Alder Hey Children's Hospital, Liverpool, UK

5: NIHR Alder Hey Clinical Research Facility, Alder Hey Children's Hospital, Liverpool, UK

Corresponding Author:

Dr. Dan Hawcutt
Alder Hey Children's Hospital
East Prescot Road
Liverpool
L14 5AB
Email: dhawcutt@liverpool.ac.uk

Word Count: 2768

Tables: 4

Figures: 2

Keywords: Montelukast, Deprescribing, Asthma, Paediatric, Systematic Review

For peer review only - http://bmjopen.bmj.com/site/about/guidelines.xhtml

Abstract

Background:

National and international asthma guidelines recommend adjusting asthma treatment based on levels of control, yet no guidance is given regarding the stepping-down of montelukast in children and young people (CYP).

Objective:

To systematically review evidence regarding deprescribing montelukast in CYP with established asthma.

Design:

Systematic review: PROSPERO ID: CRD42020213971

Data Sources:

Embase, Medline, PubMed and CINAHL were searched up to October 2020.

Study Selection:

Eligible studies contained patients aged 0-18 years with a diagnosis of asthma, who had been administering montelukast before it was withdrawn. All reasons for withdrawal were included.

Results:

The search identified 197 papers. After deduplication, five papers were included (three randomised control studies and two cohort studies). Four studies observed the impact of montelukast withdrawal for two weeks, and one study for eight weeks. The impact of withdrawal was measured in the studies using a combination of lung tests (e.g. FEV1, FeNO), asthma scoring methods and exercise challenges. Of the 17 domains in the Core Outcome Set for Clinical Trials in Childhood Asthma, eight outcomes were measured in at least one of the five studies, with all five studies measuring the outcome of 'Lung Function'. No significant differences were found between the montelukast and placebo groups following montelukast withdrawal. Significant differences between the comparator points within the test group were found in nine outcomes across four studies; FEV1/FVC, FEV1, FEF (25%-75%), asthma score (study specific), maximum % fall in FEV1 and time to recovery (post exercise) significantly decreased whereas FEV1/BDR, FeNO and eNO significantly increased.

Conclusion:

Only limited, contradictory and short-term effects of deprescribing montelukast in CYP with established asthma are presented in literature. Definitive studies determining clinical stability, and impact of deprescribing montelukast in CYP are imperative to improve the safety of asthma treatment in CYP.

For peer review only

Strengths and limitations of this study

Strengths

- With montelukast commonly used globally, this review did not limit publication eligibility based on language.
- This systematic review was conducted in accordance with PRISMA guidelines and therefore regarded as methodologically solid by healthcare professionals (audience).

Limitations

- Some papers had to be excluded due to uncertainties regarding patients' formal asthma diagnosis.

Introduction:

Asthma is a disease of lung inflammation and small airway constriction, and affects more than 338 million people globally [1]. It is the most common chronic disease in children and young people (CYP) [2]. Asthma can be of variable severity, with symptoms induced by a range of factors such as exercise, viruses or pollen [3]. As a result, the dose and class of asthma medication hugely varies between individuals in order to ensure an effective, personalised treatment plan. This rationale is encouraged by national and international guidelines which aid clinicians in their decision-making [4-8]. However, there are some ambiguities in the current guidelines.

One drug included in most guidelines is montelukast, the 16th most prescribed medication globally in 2020 [9]. Although sometimes prescribed as a first-line treatment, montelukast, a leukotriene receptor antagonist, is commonly prescribed as an additional therapy for patients whose asthma is not controlled by inhaled corticosteroids; its use therefore depends on the needs of a patient at a particular time [7 10]. While the addition and stepping down of treatment is encouraged by asthma guidelines, the process of deprescribing montelukast is not clearly described [4-8]. There is clarity about when montelukast is ineffective, where guidelines state that montelukast treatment should be stopped after an initial trial period [5-7]. However, no montelukast-specific guidance is given for the deprescribing of this drug following the achievement of "good asthma control"- the definition of which is poorly defined [4]. This is a possible consequence of the lack of data regarding the deprescribing of montelukast in literature [11].

This systematic review aims to collate the current knowledge base around the deprescribing of montelukast in CYP with asthma. The primary aim was to identify the impact of montelukast withdrawal on paediatric patients' asthma symptoms and control using the Core Outcome Set for Clinical Trials in Childhood Asthma (COS). The longevity in which the impact of montelukast withdrawal was examined for in the literature was also reviewed.

Methods:

The protocol for this review was registered in the International Prospective Register of Systematic Reviews (PROSPERO 2020: CRD42020213971) at the start of the study (September 2020). Our systematic review is reported in line with the Preferred Reporting Items for Systematic Reviews (PRISMA) guidelines.

For peer review only - http://bmjopen.bmj.com/site/about/guidelines.xhtml

Eligibility Criteria

Eligible studies contained patients aged 0-18 years with a diagnosis of asthma and to whom montelukast had been administered before it was withdrawn. Studies which contained both adult (≥18 years) and paediatric data were included if the relevant data (information regarding the deprescribing on montelukast) were recorded separately from the adult data. Human studies in any language and with any publication date were included. All primary research study designs including randomised control trials (RCT) and cohort studies were eligible. Case reports were also included to ensure patient experience regarding the deprescribing of montelukast was included. No narrative reviews or editorials were included. The primary objective was to identify the impact of montelukast withdrawal on a patient's asthma symptoms and control (including lung function, inflammation, etc) using the COS [12]. The secondary objective was to review the time over which the withdrawal of montelukast in CYP was measured.

Search Strategy and Study Selection

In September 2020, we searched Medline, PubMed, EMBASE and CINAHL using a combination of MeSH and free text subject headings, where appropriate, to include the research question (see Table S1 - Supplementary Data Section- for complete search strategy). The primary author (ED) screened the titles and abstracts of all identified studies using the eligibility criteria. Eligible studies were additionally screened using the full text. This process was repeated independently by the second author (CK) in October 2020. Subsequently the authors selected the eligible studies; a separate author (DH) independently resolved disagreements between the authors at the full text stage. Reference lists of eligible papers were manually screened for additional papers.

Patient and Public Involvement

The Paediatric Pharmacology team were awarded an NIHR Research Design Service (RDS) public involvement grant and used this to ascertain input from both the Young Persons Advisory Group (YPAG) as well as a group of young people with asthma based at Alder Hey Hospital about research around the deprescribing of montelukast. The groups were supportive of research into montelukast deprescribing in CYP, and further understanding of this issue.

Quality assessment

RTCs were appraised using the Cochrane Revised Collaboration's Risk of Bias Tool [13] and cohort studies using The Newcastle-Ottawa Quality Assessment Form [14] (Tables S2-S3- Supplementary Data Section). This was conducted independently by two authors (ED and DH). There were no discrepancies.

Data extraction and synthesis

Data were extracted by two authors independently (ED and DH). From each study, specific data were extracted related to the domains detailed in the COS (including the impact of montelukast withdrawal on asthma exacerbations, lung function and inflammation, and quality of life [12]. Results were additionally categorised into short-term (0-6 weeks), medium term (6 weeks to 6 months), and long-term effects (>6 months).

Statistical methods

Results are collated and reported descriptively. Meta-analysis was not appropriate.

For peer review only - http://bmjopen.bmj.com/site/about/guidelines.xhtml

Results

After duplicates were removed, the search identified 197 papers. We excluded 182 papers based upon the title and abstract and a further 10 following full-text screening. Five papers met the eligibility criteria (Fig. 1). The eligible papers comprised of three RCT and two cohort studies. In total 155 patients were included, of which 107 were both administered, and withdrawn from, montelukast. Further characteristics of the studies are presented in Table 1. The design of each study including montelukast administration and withdrawal is shown in Fig. 2

For peer review only

Table 1: Eligible studies| Details of the studies which met the eligibility criteria.

Study						Patients				
Name of Study	Authors	Date	Study Type	Study Design	Patients Who Completed the Study	Patients Given Montelukast (n)	Age of Patients (years)	Asthma Status Inclusion Criteria	Dose of Montelukast (mg)	Other Drugs Permited During Study
Airway mechanics after withdrawal of a leukotriene receptor antagonist in children with mild persistent asthma: Double-blind, randomized, cross-over study	Kim J.H.; Lee S.; Han M.Y. *et al.*	2020	Randomised, double-blind, placebo controlled, cross-over study	Placebo or montelukast for 2 weeks, washout for 1 week and swap treatment group for 2 weeks.	28	28	7.1 (mean)	1) On Global Initiative for Asthma guidelines step-2 treatment with Montelukast for 1 month or longer. 2) Absence of asthma exacerbations or respiratory symptoms at least 1 month before study onset. 3) No use of systemic corticosteroids during the previous 4 weeks.	4 or 5	Short-acting bronchodilator
Exhaled nitric oxide before and after montelukast sodium therapy in school-age children with chronic asthma: A preliminary study	Bratton D.L.; Lanz M.J.; Miyazawa N. *et al.*	1999	Cohort study	2 weeks run-in, treatment for 4 weeks, 2-week washout. Controls (matched by age and gender, no history of asthma) were given no placebo.	24	12	6- 11	1) Clinical history of mild to moderate stable asthma. 2) Presence of symptoms requiring beta-agonist therapy on at least 7 of the 14 days ruing the run-in period with evidence of airway reversibility (> 12% improvement in FEV1). 3) Judged to be in good health on the basis of history and physical examination.	5	Constant dose of nasal corticosteroids

For peer review only - http://bmjopen.bmj.com/site/about/guidelines.xhtml

Effects of montelukast treatment and withdrawal on fractional exhaled nitric oxide and lung function in children with asthma	Montuschi P.; Mondino C.; Koch P. *et al.*	2007	Randomised, double-blind, placebo controlled, parallel group study	1 week run-in, treatment or placebo for 4 weeks, 2 weeks washout.	26	14	10.5 (mean, placebo)<br><br>10.8 (mean, treatment)	1) Have mild persistent asthma defined by the National Heart, Lung and Blood Institute of the National Institutes of Health.<br>2) Have symptoms more often than twice a week but less often than once a day.<br>3) FEV1 > 80% of predicted value and reversibility of > 12 % to salbutamol or a positive provocation test result with methacholine or exercise.<br>4) No leukotriene receptor antagonist in the previous 4 weeks.<br>5) No glucocorticoids for > 4 weeks in previous year.	5	Short-acting bronchodilator
Effects of montelukast on symptoms and eNO in children with mild to moderate asthma	Lee M.Y.; Lai Y.S.; Yang K.D. *et al.*	2005	Cohort study	2 weeks run-in, treatment for 2 weeks, 2 weeks washout.	13	13	6- 14	1) Have mild to moderate persistent asthma as defined by the National Asthma Education and Prevention Program, Expert Panel Report II, Guidelines for the Diagnosis and Management of Asthma.<br>2) No corticosteroids or leukotriene D4 receptor antagonist treatment in the previous 4 weeks.	5	Short-acting bronchodilator
Prolonged effect of montelukast in asthmatic children with exercise-induced bronchoconstriction	Kim J.H.; Lee S.Y.; Kim H.B. *et al.*	2004	Randomised, parallel-group study	Period 1: Treatment or placebo for 8 weeks (double blind). Period 2: Groups unblinded. Treatment group re-randomised for 8 weeks washout or treatment (single blind).	64	Period 1: 40 Period 2: 12	8- 14 (mean, 10.3 treatment)<br><br>(10.4 mean, placebo)	1) Have mild asthma defined by the American Thoracic Society.<br>2) History of exercise-induced bronchoconstriction.<br>3) FEV1 of > 80% predicted and a decrease of >15 % after standardised exercise challenge at screening.<br>4) No corticosteroids, long-acting beta-agonists or leukotriene receptor antagonists.	5	Short-acting bronchodilator

For peer review only

Risk of Bias of Included Studies

Tables S2-S3 show the results of the risk of bias assessments for the five papers examined. Both cohort studies show an overall low risk of bias, but one (Lee *et al.,* 2005) demonstrated some uncertainly due to the selection of the non-exposed cohort (no control group) [15 16]. The RCTs were judged to have low risk [17-19].

Montelukast Withdrawal: Comparator Points

The studies conducted by Bratton *et al.,* Montuschi *et al.* and Lee *et al.* all measured the impact of montelukast withdrawal using their chosen criteria by comparing asthma symptoms on the final day of montelukast treatment to those on the final day of the washout period (Fig. 2). Montuschi *et al.* further compared the treatment group to a placebo group with Bratton *et al.* using match controls (no-placebo administered). Lee *et al.* did not use a placebo or control group.

Kim *et al.* (2004) measured the impact of montelukast withdrawal by comparing the asthma symptoms of the 'Period One' treatment group on the final day of montelukast administration (end of week eight) to the asthma symptoms of the 'Period Two' placebo group on the final day of placebo administration (end of week 16). As illustrated in Fig. 2, at the end of 'Period One', the participants were un-blinded, and the placebo group participants were removed from the study. The 'Period One' montelukast group was subsequently re-randomised for 'Period Two'.

Kim *et al.* (2020) measured the impact of montelukast withdrawal by comparing asthma symptoms at baseline (week 1) with those following placebo administration (week three or week six) (Fig. 2). It should be noted that montelukast administration for ≥ one month was an inclusion criteria in Kim *et al.*'s study design and therefore by comparing those participants taking placebo to the baseline, the impact of deprescribing is being measured. The same comparisons were also made following montelukast administration (week three or week six) (Fig. 2).

Core Outcomes Set

Across the five eligible studies, the impact of withdrawing montelukast from children with asthma was only measured against eight of the 17 COS (Table 2). A total of 20 outcomes, measured using 13 unique measures, were used to quantify change in individuals before and after montelukast was withdrawn. No data regarding the COS of death, growth, long term health-related problems, long-term adverse effect, ability to carry out "normal activities", quality of life, school attendance, general practise (GP)/ accident and emergency (A + E) attendance and hospital admission were presented in any of the five studies.

For peer review only

Table 2: Presence of Core Outcome Set in Studies

Domains of COS	Core Outcome Set for School-Aged Children with Asthma	Kim *et al.*, 2020	Bratton *et al.*, 1999	Montuschi *et al.*, 2007	Lee *et al.*, 2005	Kim *et al.*, 2004
**Symptom Control**	Daytime Symptoms	+*	-	-	+*	+*
	Death	-	-	-	-	-
	Exacerbations	+*	-	-	+*	-
	Lung Tests	+	+	+	+	+
	Nocturnal Symptoms	+*	-	-	+*	+*
	Parent/ Child Global Assessment of Control	+	-	-	-	-
	Use of Reliever Inhaler	-	+	-	+	-
**General Health Impact**	Growth	-	-	-	-	-
	Health-Related Problems when Older	-	-	-	-	-
	Long-Term Adverse Effect	-	-	-	-	-
	Short-Term Adverse Effect	-	-	-	+	+
**Healthcare Required**	GP/ A + E Attendance	-	-	-	-	-
	Hospital Admission	-	-	-	-	-
**Life Factors**	Activity or Exercise	+*	-	-	-	+
	Normal Activities	-	-	-	-	-
	Quality of Life	-	-	-	-	-
	School Attendance	-	-	-	-	-

**Parameter included in asthma severity score. Full details of all asthma severity scores were not detailed in the studies.*

Exacerbation

Kim, *et al* [18] used the Child Asthma Control Test (C-ACT) questionnaire to assess asthma control in both the placebo and test group [20]. No significant difference was found between changes in C-ACT score between treatment groups or the comparator points. Lee, *et al* [16] also used an asthma scoring system which monitored factors including asthma exacerbations. Asthma scores did not significantly change during the two-week washout period post montelukast administration (weeks four to six- Fig. 2). Full details of this asthma scoring system were not able to be identified. In addition, Lee, *et al* [16] recorded that two of the 13 patients experienced mild asthma attacks during this two-week period, demonstrating a worsening of asthma control (Table 3).

Daytime and Nocturnal Symptoms

The C-ACT questionnaire used by Kim, *et al* [18] included an assessment of daytime and nocturnal symptoms [20]. No significant difference was found between changes in C-ACT score between the placebo and test groups or the comparator points [18]. The asthma scoring systems used by Lee, *et al* [16] also described monitoring factors including daytime and nocturnal symptoms. The asthma score did not significantly change during the washout period (weeks four to six- Fig. 2). However, between weeks eight and 16 (Fig. 2), there was a significant decrease ($p < 0.05$) in the asthma score (improved symptom control) of the Period 2 placebo group used by Kim, *et al* [17], which accounted for daytime

For peer review only - http://bmjopen.bmj.com/site/about/guidelines.xhtml

and night-time asthma symptoms (Table 4). Full details of this asthma scoring system were not able to be identified [17].

Activity or exercise

The C-ACT questionnaire used by Kim, *et al* [18] included an assessment of exercise [20]. No significant difference was found between changes in C-ACT score between the placebo and test groups or between the comparator points. However, in the study conducted by Kim, *et al* [17] the time taken for a patient's Forced Expiratory Volume in one second (FEV1) to return within 10% of their pre-exercise baseline following a standardised exercise challenge significantly decreased (p <0.050) between the end of montelukast administration and the end of the washout period (weeks eight and 16 of the Period 2 placebo group- Fig. 2), demonstrating an improvement in asthma control (Table 4).

Short-term adverse effects

Reports of a mild headache subsisting spontaneously two days after montelukast withdrawal were recorded by Lee, *et al* [16].

Lung Tests

All five studies undertook lung function tests to assess the impact of montelukast withdrawal on lung function.

The change in FEV1 between comparator points was measured in two of the five studies (Table 3) [18] [19]. Montuschi *et al.* [19] recorded a significant decrease (p=0.011, Table 4) in FEV1 between the final day of montelukast administration and the end of the washout period (Fig. 2) demonstrating a reduced pulmonary function (worsening of asthma symptoms) following montelukast withdrawal (Table 4). However, Kim *et al.* [18] recorded no significant change.

Both Kim *et al.* [18] and Montuschi *et al.* [19] recorded a significant decrease (p=0.03 and p<0.003, respectively) in FEV1/ Forced Vital Capacity (FVC) values (Table 4) between comparator points (Fig. 2), demonstrating a worsening of asthma symptoms. This was further shown by Kim *et al.* [18] through the significant increase (p=0.04) in FEV1/ Bronchodilator Response (BDR) between comparator points (Fig. 2), demonstrating an increase in bronchial hyper-responsiveness following montelukast withdrawal (Table 4). Montuschi *et al.* [19] also described that Forced Expiratory Flows (FEF) (25%-75%) significantly decreased (p<0.03) between comparator points (Fig. 2).

Montuschi *et al.*[19] recorded a significant increase (p=0.023) in Fractional Exhaled Nitric Oxide (FeNO) following the washout phase (weeks five to seven- Fig. 2), demonstrating an increase in eosinophilic airway inflammation (Table 3 and Table 4). Changes in Exhaled Nitric Oxide (eNO) recorded by Lee *et al.* [16] also demonstrate a reduced pulmonary function (significant increase, p=0.011) during the washout period (Fig. 2, Table 4) however Bratton *et al.* [15] recorded a non-significant change for the same measure (Table 3).

There were no significant changes during the washout periods in PEFR and Impulse Oscillometry in Small Airways across any of the five studies (Table 3).

For peer review only - http://bmjopen.bmj.com/site/about/guidelines.xhtml

Table 3: Significant differences following the deprescribing of montelukast| The recorded outcomes of deprescribing montelukast in children with established asthma.

Study	Measure	Significant Difference between the Test Group and Placebo Following Montelukast Withdrawal	Significant Difference between the Test Group/ Group of Interest between the Comparator Points
Kim et al., 2020	FEV1	No	No
	FEV1/FVC		Yes
	IOS		No
	FeNO		No
	C-ACT*		No
	FEV1/BDR		Yes
Bratton *et al.*, 1999	eNO	N/A	No
Montuschi *et al.*, 2007	FeNO	No	Yes
	FEV1		Yes
	FEV1/FVC		Yes
	FEF (25%-75%)		Yes
Lee *et al.*, 2005	eNO	N/A	Yes
	PEFR		No
	Asthma Score		No
	Asthma Attack		N/A (2)
Kim *et al.*, 2004	Asthma Score	No	Yes
	Maximum % fall in FEV1		Yes
	Time to Recovery		Yes

For peer review only

Table 4: Significant differences in measures between the end of montelukast administration and the end of the washout period (Fig. 2)

Study	Measure with Significant Change Across Studies	Significant Change Between of Test Group/ Group of Interest	Value at First Comparator Point	Value at Second Comparator Point
Kim *et al.*, 2020	FEV1/FVC	Significantly decrease (p= 0.03)	86% (95% CI: 83%–89%)	83% (95% CI: 80%–86%)
	FEV1/BDR	Significantly increase (p= 0.04)	6.42 (95% CI: 3.74% - 9.11%)	10.72 (95% CI: 5.82% - 15.61%)
Montuschi *et al.*, 2007	FeNO	Significantly increased (p = 0.023)	37.9 ppb (25.6-62.5) (median (interquartile range))	52.2 ppb (36.0-72.9) (median (interquartile range))
	FEV1	Significantly decreased (p = 0.011)	2.48 L ± 0.22 (mean ± SEM)	2.33 ± 0.19 (mean ± SEM)
	FEV1/FVC	Significantly decreased (p ≤ 0.003)	Not stated in text-graphically presented only	Not stated in text-graphically presented only
	FEF (25%-75%)	Significantly decreased (p ≤ 0.03)	Not stated in text-graphically presented only	Not stated in text-graphically presented only
Lee *et al.*, 2005	eNO	Significantly increased (p = 0.011)	13.5 ppb ± 7.60 (mean ± SD)	29.2 ppb ± 16.1 (mean ± SD)
Kim *et al.*, 2004	Asthma Score	Significantly decreased (p <0.050)	17.8 ± 6.8 (mean ± SD)	17.7 ± 6.78 (mean ± SD)
	Maximum % fall in FEV1	Significantly decreased (p <0.050)	27.6% ± 14.4 (mean ± SD)	26.7 % ± 19.4 (mean ± SD)
	Time to Recovery	Significantly decreased (p <0.050)	25.3 mins ± 23.3 (mean ± SD)	27.7 mins ± 26.5 (mean ± SD)

Timeframe

Four studies [15 16 18 19] observed the impact of montelukast withdrawal (a washout period) for two weeks (short-term) and one study [17] for eight weeks (medium-term). No study examined the long-term effects (>6 months) of montelukast withdrawal.

Discussion

Interest in stepping down montelukast has heightened since the FDA applied a boxed warning to montelukast in March 2020 due to the increasing number of neuropsychiatric events reported [21 22].

For peer review only - http://bmjopen.bmj.com/site/about/guidelines.xhtml

Since then, further concerns regarding the adverse drug reactions attributed to montelukast in CYP with asthma have been outlined in a recent PROSPERO systematic review [23].

Despite these, this review highlights the limited number of studies which have formally examined the stepping down from montelukast in CYP with diagnosed asthma. Within identified studies, only 119 children and young people have been included, split between a range of methodologies, all of which limit clinical interpretation and implementation. The five eligible papers primarily examine lung function as a proxy for asthma. Measuring lung function is a commonplace technique used to understand and aid the diagnosis of asthma. However, for most methods of measuring lung function, there is very little information about how the measure relates to asthma management in children in RCT or large longitudinal studies [24]. For example, in 2017 Murray *et al.* concluded that the diagnostic algorithm, which uses data from FEV1, FeNO and FVC measurements, was inaccurate [25]. Therefore, the use of lung function measures, of which make up 14 of the 18 measures undertaken across the eligible studies, do not significantly help with the clinical understanding of the impact of montelukast withdrawal in children with asthma.

Additionally, there were few domains from the relevant core outcomes captured and examined. There was only one study that aligned their outcomes with the COS, examining a maximum of six of the 17 domains (Table 2) [12].

Lastly, the studies examine the effect of the withdrawal of montelukast over a short time frame relative to the expected length of treatment. With four of the five studies only observing the patients for two week post final montelukast administration, only some aspects of the impact deprescribing montelukast has on patients in the immediate post-administration phase have been examined. As a result, the only knowledge found in literature regarding the effects of deprescribing montelukast for a period greater than two weeks comes from 64 paediatric asthma patients in a single study [17]. Further studies examining the deprescribing of montelukast in CYP with asthma over longer time periods to capture events like effects of seasonality, growth, and longer term school attendance and attainment (with regard to known neuropsychiatric adverse effects) are necessary.

Strengths and Limitations of this Study

This is the first systematic review of deprescribing montelukast in paediatric asthma patients, and while limited data were identified, this provides a clear direction in terms of the outcomes that need to be captured (improved alignment with the COS), and the study designs (e.g. longer time frames) in future research.

However, a limitation of this review is the unknown contents of the two asthma scoring systems used by Lee *et al.* and Kim *et al.* [16 17]. Although some of the factors included in these scores are listed in their publications (e.g. night-time symptoms) and therefore included in Table 2, the extent of the scores could not be examined as part of this review. It is possible that certain factors outlined in by the COS for trials of childhood asthma were examined as part of the scoring systems used. However, these were small studies, comprising only 41 paediatric asthma patients in total, and even if a large number of domains with the COS were captured, it is unlikely to have affected our view that the impact on children with asthma is not well described. Additionally, it is possible that individual factors examined in the scoring systems did significantly change between comparator points, but the overall asthma score did not and therefore the change in the factor was masked.

For peer review only - http://bmjopen.bmj.com/site/about/guidelines.xhtml

Conclusion

To conclude, the knowledge regarding the impact of deprescribing montelukast in children with asthma in relation to the Core Outcome Set for Clinical Trials in Childhood Asthma (COS) [12] in literature is limited, contradictory, and only the short-term effects of stepping down this therapy are known. Definitive studies determining clinical stability, and impact of deprescribing montelukast in CYO are imperative in order for guidelines to fully reflect the overall impact of stepping down this treatment in children.

Funding

This study did not receive specific funding.

Conflict of Interests

No conflicts of interest were declared.

Acknowledgements

This is a summary of independent research carried out at the National Institute for Health Research (NIHR) Alder Hey Clinical Research Facility. The views expressed are those of the author(s) and not necessarily those of the NHS, the NIHR or the Department of Health.

Figure Legends

Figure 1: Search strategy

Figure 2: The design of the eligible studies| Where the participants in studies were split into treatment groups, each group is shown in parallel.

Contributorship

ED conducted the systematic review. This included establishing the eligibility criteria, conducting the search, collecting the data and analysing the results. CK conducted the data search in parallel and compared eligible papers with ED. AL advised and provided information on deprescribing and prescribing policy and guidelines. IS and DH equally oversaw the direction of the review including editing the writing of the paper and acting as consultants in ensuring clinical relevance of the review.

Data Availability

The data used in this review was collected from the five eligible studies and therefore available in the public domain.

Ethical Approval Statement

Not applicable

For peer review only - http://bmjopen.bmj.com/site/about/guidelines.xhtml

References

1. Ferrante G, La Grutta S. The Burden of Pediatric Asthma. Frontiers in Pediatrics 2018;6(186) doi: 10.3389/fped.2018.00186

2. World Health Organisation. Asthma: World Health Organisation,; 2020 [updated 15/05/2020. Available from: https://www.who.int/news-room/q-a-detail/asthma accessed 09/09/2020 2020.

3. Colice GL. Categorizing asthma severity: an overview of national guidelines. Clin Med Res 2004;2(3):155-63. doi: 10.3121/cmr.2.3.155

4. Global Initiative for Athma. Global Strategy for Asthma Management and Prevention, 2021.

5. National Asthma Council Australia. Australian Asthma Handbook, Quick Reserence Guide 2014 [Available from: http://www.trmc.net.au/pdf/australian-asthma-handbook.pdf accessed 03/03/2021 2021.

6. National Institute for Health and Care Excellence. Asthma: diagnosis, monitoring and chronic asthma management 2017 [Available from: https://www.nice.org.uk/guidance/ng80/resources/asthma-diagnosis-monitoring-and-chronic-asthma-management-pdf-1837687975621 accessed 03/02/21 2021.

7. British Thoracic Society and The Scottish Intercollegiate Guidelines Network. SIGN158 British Guidelines on the Management of Asthma [Clinical Guidelines]. 2019 [updated July 2020; cited 2020 09/09/2020]. Available from: https://www.brit-thoracic.org.uk/quality-improvement/guidelines/asthma/ accessed 09/09/2020 2020.

8. Chung KF, Wenzel SE, Brozek JL, et al. International ERS/ATS guidelines on definition, evaluation and treatment of severe asthma. European Respiratory Journal 2014;43(2):343-73. doi: 10.1183/09031936.00202013

9. OpenPrescribing. Montelukast (0303020G0) 2020 [updated 01/06/2020. Available from: https://openprescribing.net/chemical/0303020G0/ accessed 14/09/2020 2020.

10. Tamada T, Ichinose M. Leukotriene Receptor Antagonists and Antiallergy Drugs. Handbook Exp Pharmacol 2017;237:153-69. doi: 10.1007/164_2016_72 [published Online First: 2016/11/09]

11. King C, Dixon EG, Hawcutt DB. Editorial: Stepping down asthma treatment in children. Pediatric Pulmonology 2021;https://doi.org/10.1002/ppul.25410 doi: https://doi.org/10.1002/ppul.25410

12. Sinha IP, Gallagher R, Williamson PR, et al. Development of a core outcome set for clinical trials in childhood asthma: a survey of clinicians, parents, and young people. Trials 2012;13(1):103. doi: 10.1186/1745-6215-13-103

13. Julian PT Higgins, Jelena Savović MJP, Jonathan AC Sterne. The Cochrane Collaboration’s tool for assessing risk of bias in randomised trials 2019 [updated 22/08/2019. Available from: https://methods.cochrane.org/bias/resources/rob-2-revised-cochrane-risk-bias-tool-randomized-trials accessed 22/09/2021 2021.

14. Wells G, Shea B, O'Connell D, et al. The Newcastle-Ottawa Scale (NOS) for assessing the quality of nonrandomised studies in meta-analyses: The Ottawa Hospital Research Institute; 2019 [Available from: http://www.ohri.ca/programs/clinical_epidemiology/oxford.asp accessed 08/12/2020 2020.

For peer review only - http://bmjopen.bmj.com/site/about/guidelines.xhtml

[dataset] [15] Bratton DL, Lanz MJ, Miyazawa N, et al. Data from: Exhaled nitric oxide before and after montelukast sodium therapy in school-age children with chronic asthma: a preliminary study. Pediatr Pulmonol, December 1999. doi: 10.1002/(sici)1099-0496(199912)28:6<402::aid-ppul3>3.0.co;2-v

[dataset] [16] Lee M-Y, Lai Y-S, Yang KD, et al. Data from: Effects of montelukast on symptoms and eNO in children with mild to moderate asthma. Pediatrics International, November 21, 2005. doi: 10.1111/j.1442-200x.2005.02142.x

[dataset] [17] Kim J-H, Lee S-Y, Kim H-B, et al. Data from: Prolonged effect of montelukast in asthmatic children with exercise-induced bronchoconstriction. Pediatric Pulmonology, September 13, 2004. doi: 10.1002/ppul.20156

[dataset] [18] Kim JH, Lee S, Shin YH, et al. Data from: Airway mechanics after withdrawal of a leukotriene receptor antagonist in children with mild persistent asthma: Double-blind, randomized, cross-over study. Pediatric Pulmonology, September 17, 2020. doi: https://doi.org/10.1002/ppul.25085

[dataset] [19] Montuschi P, Mondino C, Koch P, et al. Effects of montelukast treatment and withdrawal on fractional exhaled nitric oxide and lung function in children with asthma. Chest, December, 2007. doi: 10.1378/chest.07-1587

20. Nathan RA, Sorkness CA, Kosinski M, et al. Childhood Asthma Control Test for Children 4 to 11 Years. 2004 [Available from: https://www.greenhillspeds.com/wp-content/uploads/2015/12/Asthma-Control-Test-4-to-11-years.pdf.

21. U.S. Food and Drug Administration. FDA Requires Stronger Warning About Risk of Neuropsychiatric Events Associated with Asthma and Allergy Medication Singulair and Generic Montelukast, 2020.

22. E.J. MUNDELL. Allergy Med Singulair to Get 'Black Box' Warning Over Psych Side Effects: FDA. US News 2020.

23. Eleanor G Dixon, Charlotte Rugg- Gunn, Vanessa Sellick, et al. Adverse Drug Reactions of Leukotriene Receptor Antagonists in Children with Asthma: A Systematic Review. British Medical Journal Paediatrics Open 2021;IN PRESS(IN PRESS)

24. Moeller A, Carlsen K-H, Sly PD, et al. Monitoring asthma in childhood: lung function, bronchial responsiveness and inflammation. European Respiratory Review 2015;24(136):204-15. doi: 10.1183/16000617.00003914

25. Murray C, Foden P, Lowe L, et al. Diagnosis of asthma in symptomatic children based on measures of lung function: an analysis of data from a population-based birth cohort study. The Lancet Child & Adolescent Health 2017;1(2):114-23. doi: 10.1016/s2352-4642(17)30008-1

1. Ferrante G, La Grutta S. The Burden of Pediatric Asthma. *Frontiers in Pediatrics* 2018;6(186) doi: 10.3389/fped.2018.00186
2. World Health Organisation. Asthma: World Health Organisation,; 2020 [updated 15/05/2020. Available from: https://www.who.int/news-room/q-a-detail/asthma accessed 09/09/2020 2020.

For peer review only

For peer review only - http://bmjopen.bmj.com/site/about/guidelines.xhtml

3. Colice GL. Categorizing asthma severity: an overview of national guidelines. *Clin Med Res* 2004;2(3):155-63. doi: 10.3121/cmr.2.3.155
4. Global Initiative for Athma. Global Strategy for Asthma Management and Prevention, 2021.
5. National Asthma Council Australia. Australian Asthma Handbook, Quick Reserence Guide 2014 [Available from: http://www.trmc.net.au/pdf/australian-asthma-handbook.pdf accessed 03/03/2021 2021.
6. National Institute for Health and Care Excellence. Asthma: diagnosis, monitoring and chronic asthma management 2017 [Available from: https://www.nice.org.uk/guidance/ng80/resources/asthma-diagnosis-monitoring-and-chronic-asthma-management-pdf-1837687975621 accessed 03/02/21 2021.
7. British Thoracic Society and The Scottish Intercollegiate Guidelines Network. SIGN158 British Guidelines on the Management of Asthma [Clinical Guidelines]. 2019 [updated July 2020; cited 2020 09/09/2020]. Available from: https://www.brit-thoracic.org.uk/quality-improvement/guidelines/asthma/ accessed 09/09/2020 2020.
8. Chung KF, Wenzel SE, Brozek JL, et al. International ERS/ATS guidelines on definition, evaluation and treatment of severe asthma. *European Respiratory Journal* 2014;43(2):343-73. doi: 10.1183/09031936.00202013
9. OpenPrescribing. Montelukast (0303020G0) 2020 [updated 01/06/2020. Available from: https://openprescribing.net/chemical/0303020G0/ accessed 14/09/2020 2020.
10. Tamada T, Ichinose M. Leukotriene Receptor Antagonists and Antiallergy Drugs. *Handbook Exp Pharmacol* 2017;237:153-69. doi: 10.1007/164_2016_72 [published Online First: 2016/11/09]
11. King C, Dixon EG, Hawcutt DB. Editorial: Stepping down asthma treatment in children. *Pediatric Pulmonology* 2021;https://doi.org/10.1002/ppul.25410 doi: https://doi.org/10.1002/ppul.25410
12. Sinha IP, Gallagher R, Williamson PR, et al. Development of a core outcome set for clinical trials in childhood asthma: a survey of clinicians, parents, and young people. *Trials* 2012;13(1):103. doi: 10.1186/1745-6215-13-103
13. Julian PT Higgins, Jelena Savović MJP, Jonathan AC Sterne. The Cochrane Collaboration's tool for assessing risk of bias in randomised trials 2019 [updated 22/08/2019. Available from: https://methods.cochrane.org/bias/resources/rob-2-revised-cochrane-risk-bias-tool-randomized-trials accessed 22/09/2021 2021.
14. Wells G, Shea B, O'Connell D, et al. The Newcastle-Ottawa Scale (NOS) for assessing the quality of nonrandomised studies in meta-analyses: The Ottawa Hospital Research Institute; 2019 [Available from: http://www.ohri.ca/programs/clinical_epidemiology/oxford.asp accessed 08/12/2020 2020.
15. Bratton DL, Lanz MJ, Miyazawa N, et al. Exhaled nitric oxide before and after montelukast sodium therapy in school-age children with chronic asthma: a preliminary study. *Pediatr Pulmonol* 1999;28(6):402-7. doi: 10.1002/(sici)1099-0496(199912)28:6<402::aid-ppul3>3.0.co;2-v [published Online First: 1999/12/10]
16. Lee M-Y, Lai Y-S, Yang KD, et al. Effects of montelukast on symptoms and eNO in children with mild to moderate asthma. *Pediatrics International* 2005;47(6):622-26. doi: 10.1111/j.1442-200x.2005.02142.x
17. Kim J-H, Lee S-Y, Kim H-B, et al. Prolonged effect of montelukast in asthmatic children with exercise-induced bronchoconstriction. *Pediatric Pulmonology* 2004;39(2):162-66. doi: 10.1002/ppul.20156
18. Kim JH, Lee S, Shin YH, et al. Airway mechanics after withdrawal of a leukotriene receptor antagonist in children with mild persistent asthma: Double-blind, randomized, cross-over study. *Pediatric Pulmonology* 2020;55(12):3279-86. doi: https://doi.org/10.1002/ppul.25085

For peer review only

For peer review only - http://bmjopen.bmj.com/site/about/guidelines.xhtml

19. Montuschi P, Mondino C, Koch P, et al. Effects of montelukast treatment and withdrawal on fractional exhaled nitric oxide and lung function in children with asthma. *Chest* 2007;132(6):1876-81. doi: 10.1378/chest.07-1587
20. Nathan RA, Sorkness CA, Kosinski M, et al. Childhood Asthma Control Test for Children 4 to 11 Years. 2004 [Available from: https://www.greenhillspeds.com/wp-content/uploads/2015/12/Asthma-Control-Test-4-to-11-years.pdf.
21. U.S. Food and Drug Administration. FDA Requires Stronger Warning About Risk of Neuropsychiatric Events Associated with Asthma and Allergy Medication Singulair and Generic Montelukast, 2020.
22. E.J. MUNDELL. Allergy Med Singulair to Get 'Black Box' Warning Over Psych Side Effects: FDA. *US News* 2020.
23. Eleanor G Dixon, Charlotte Rugg- Gunn, Vanessa Sellick, et al. Adverse Drug Reactions of Leukotriene Receptor Antagonists in Children with Asthma: A Systematic Review. *British Medical Journal Paediatrics Open* 2021;IN PRESS(IN PRESS)
24. Moeller A, Carlsen K-H, Sly PD, et al. Monitoring asthma in childhood: lung function, bronchial responsiveness and inflammation. *European Respiratory Review* 2015;24(136):204-15. doi: 10.1183/16000617.00003914
25. Murray C, Foden P, Lowe L, et al. Diagnosis of asthma in symptomatic children based on measures of lung function: an analysis of data from a population-based birth cohort study. *The Lancet Child & Adolescent Health* 2017;1(2):114-23. doi: 10.1016/s2352-4642(17)30008-1

For peer review only

For peer review only - http://bmjopen.bmj.com/site/about/guidelines.xhtml

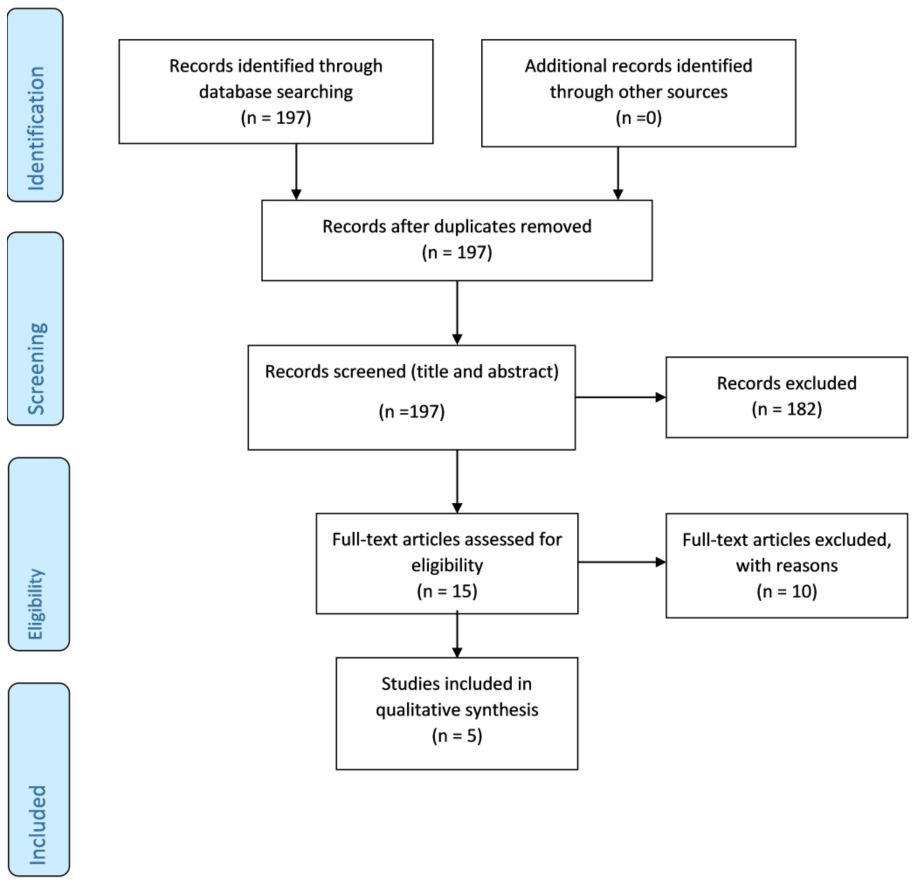

Figure 1: Collection of Sources

1233x1175mm (96 x 96 DPI)

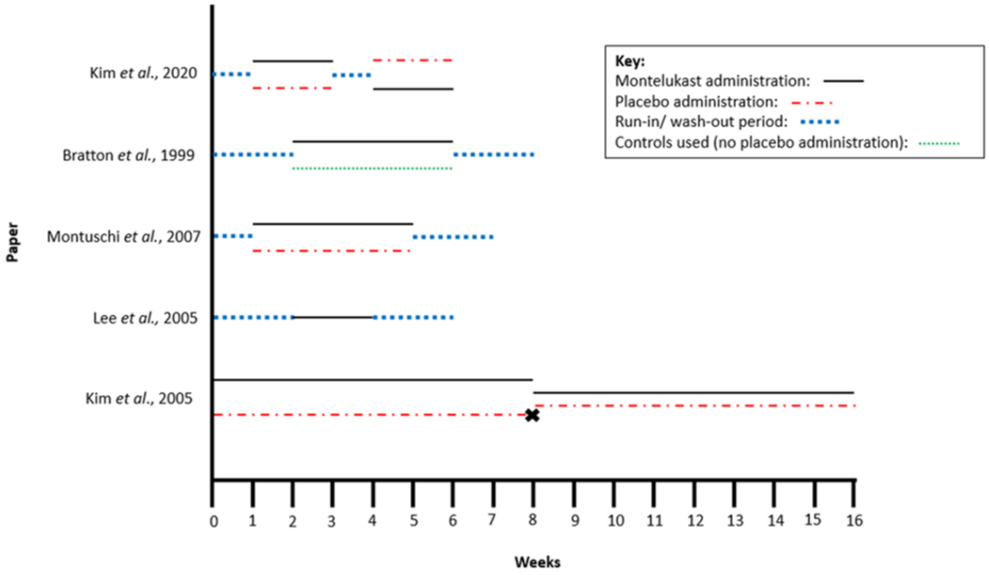

Figure 2: The design of the eligible studies| Where the participants in studies were split into treatment groups, each group is shown in parallel.

630x374mm (38 x 38 DPI)

Deprescribing Montelukast in Children with Asthma: A Systematic Review (SUPPLEMENTARY DATA SECTION)

Eleanor Dixon[1,2], Charlotte King[3], Andrew Lilley[4], Ian Sinha[5], Daniel B Hawcutt[2,4]

1: Department of Pharmacology and Therapeutics, University of Liverpool, UK

2: Department of Women's and Children's Health, University of Liverpool, UK

3: Liverpool University Hospital Trust, Liverpool, UK

4: NIHR Alder Hey Clinical Research Facility, Alder Hey Children's Hospital, Liverpool, UK

5: Department of Respiratory Medicine, Alder Hey Children's Hospital, Liverpool, UK

Tables: 3

Keywords: Montelukast, Adverse Drug Reactions, Asthma, Paediatric, Systematic Review

For peer review only

Methods

Table S1: Search Strategy

Strategy 918451 #	Database	Search term	Results
1	EMBASE	CHILD/ OR INFANT/ OR BABY/ OR NEWBORN/	2417196
2	EMBASE	JUVENILE/ OR CHILD/ OR BOY/ OR GIRL/ OR INFANT/ OR TODDLER/ OR "SCHOOL CHILD"/ OR "PRESCHOOL CHILD"/	2301714
3	EMBASE	JUVENILE/ OR ADOLESCENT/	1563364
4	EMBASE	PEDIATRICS/	78300
5	EMBASE	((Paediatric* OR Infan* OR Newborn* OR Bab* OR Neonat* OR Preterm* OR Prematur* OR postmatur* OR Child* OR School* OR "Nursery school*" OR Kindergar* OR "Primary school*" OR "Secondary school*" OR "Elementary school*" OR "High school*" OR Highschool* OR Preschool* OR Kid* OR Toddler* OR Adoles* OR Teen* OR Boy* OR Girl* Minor* OR Pubert* OR Pubescen* OR Prepubescen*) NOT REVIEW*).ti,ab	3217377
6	EMBASE	"MINOR (PERSON)"/	653
7	EMBASE	ADOLESCENCE/ OR PUBERTY/ OR PREPUBERTY/	70704
8	EMBASE	(1 OR 2 OR 3 OR 4 OR 5 OR 6 OR 7)	4839396
9	EMBASE	8 [Humans]	4025357
10	EMBASE	ASTHMA/ OR "ASTHMA BRONCHIALE"/	231772
11	EMBASE	((Asthma* OR Wheez* OR "Bronchial asthma*" OR "stable asthma") NOT REVIEW).ti,ab	216313
12	EMBASE	(10 OR 11)	284696
13	EMBASE	12 [Humans]	238534
14	EMBASE	DEPRESCRIPTION/ OR DEPRESCRIPTIONS/	501
15	EMBASE	"TREATMENT WITHDRAWAL"/ OR "DRUG WITHDRAWAL"/	215067
16	EMBASE	((deprescrib* OR stop* OR withdraw* OR ceas* OR discontinu* OR de-prescrib*) NOT REVIEW).ti,ab	536525
17	EMBASE	(14 OR 15 OR 16)	680842
18	EMBASE	17 [Humans]	519666
19	EMBASE	MONTELUKAST/	9658
20	EMBASE	((montelukast OR singular) NOT REVIEW).ti,ab	15377
21	EMBASE	(19 OR 20)	21572
22	EMBASE	21 [Humans]	14849

For peer review only

23	EMBASE	(8 AND 12 AND 17 AND 21)	194
24	EMBASE	23 [Humans]	191
25	Medline	CHILD/ OR "CHILD, PRESCHOOL"/	1916435
26	Medline	ADOLESCENT/ OR MINORS/	2036089
27	Medline	INFANT/ OR "INFANT, NEWBORN"/	1139739
28	Medline	((Paediatric* OR Infan* OR Newborn* OR Bab* OR Neonat* OR Preterm* OR Prematur* OR postmatur* OR Child* OR School* OR "Nursery school*" OR Kindergar* OR "Primary school*" OR "Secondary school*" OR "Elementary school*" OR "High school*" OR Highschool* OR Preschool* OR Kid* OR Toddler* OR Adoles* OR Teen* OR Boy* OR Girl* Minor* OR Pubert* OR Pubescen* OR Prepubescen*) NOT REVIEW*).ti,ab	2716250
29	Medline	PUBERTY/	13325
30	Medline	(25 OR 26 OR 27 OR 28 OR 29)	4733651
31	Medline	ASTHMA/	125895
32	Medline	((Asthma* OR Wheez* OR "Bronchial asthma*" OR "stable asthma") NOT REVIEW).ti,ab	155279
33	Medline	(31 OR 32)	177833
34	Medline	DEPRESCRIPTIONS/	466
35	Medline	((deprescrib* OR stop* OR withdraw* OR ceas* OR discontinu* OR de-prescrib*) NOT REVIEW).ti,ab	369364
36	Medline	(34 OR 35)	369497
37	Medline	((montelukast OR singular) NOT REVIEW).ti,ab	13162
38	Medline	(30 AND 33 AND 36 AND 37)	54
39	Medline	38 [Humans]	50
40	PubMed	((Paediatric* OR Infan* OR Newborn* OR Bab* OR Neonat* OR Preterm* OR Prematur* OR postmatur* OR Child* OR School* OR "Nursery school*" OR Kindergar* OR "Primary school*" OR "Secondary school*" OR "Elementary school*" OR "High school*" OR Highschool* OR Preschool* OR Kid* OR Toddler* OR Adoles* OR Teen* OR Boy* OR Girl* Minor* OR Pubert* OR Pubescen* OR Prepubescen*) NOT REVIEW*).ti,ab	143769
41	PubMed	((Asthma* OR Wheez* OR "Bronchial asthma*" OR "stable asthma") NOT REVIEW).ti,ab	161822
42	PubMed	((deprescrib* OR stop* OR withdraw* OR ceas* OR discontinu* OR de-prescrib*) NOT REVIEW).ti,ab	361553
43	PubMed	((montelukast OR singular) NOT REVIEW).ti,ab	13080
44	PubMed	(40 AND 41 AND 42 AND 43)	0
45	CINAHL	CHILD/ OR INFANT/ OR ADOLESCENCE/ OR "MINORS (LEGAL)"/ OR "CHILD, PRESCHOOL"/	892377
46	CINAHL	PEDIATRICS/ OR NEONATOLOGY/	20791
47	CINAHL	PUBERTY/	3060
48	CINAHL	ADOLESCENCE/ OR CHILD/ OR "MINORS (LEGAL)"/	778755
49	CINAHL	((Paediatric* OR Infan* OR Newborn* OR Bab* OR Neonat* OR Preterm* OR Prematur* OR postmatur* OR Child* OR School* OR "Nursery school*" OR Kindergar* OR "Primary school*" OR "Secondary school*" OR "Elementary school*" OR "High school*" OR Highschool* OR Preschool* OR Kid* OR Toddler* OR Adoles* OR Teen* OR Boy* OR Girl* Minor* OR Pubert* OR Pubescen* OR Prepubescen*) NOT REVIEW*).ti,ab	841196
50	CINAHL	(45 OR 46 OR 47 OR 48 OR 49)	1279403

For peer review only

51	CINAHL	ASTHMA/	34317
52	CINAHL	((Asthma* OR Wheez* OR "Bronchial asthma*" OR "stable asthma") NOT REVIEW).ti,ab	36601
53	CINAHL	((deprescrib* OR stop* OR withdraw* OR ceas* OR discontinu* OR de-prescrib*) NOT REVIEW).ti,ab	73381
54	CINAHL	(51 OR 52)	45034
55	CINAHL	MONTELUKAST/	368
56	CINAHL	((montelukast OR singular) NOT REVIEW).ti,ab	1953
57	CINAHL	(55 OR 56)	2122
58	CINAHL	(50 AND 53 AND 54 AND 57)	14

Results

Table S2: Results of assessment of risk bias of randomised control trials| The Cochrane Collaboration's tool was used. Green= low risk of bias, Yellow= unclear risk of bias, Red= high risk of bias.

	**Kim *et al.*, 2020**	**Montuschi *et al.*, 2007**	**Kim *et al.*, 2005**
Random sequence generation (randomisation bias)	Green	Yellow	Yellow
Allocation concealment (randomisation bias)	Green	Green	Green
Baseline differences (randomisation bias)	Green	Green	Green
Participant awareness (deviation bias)	Green	Green	Green
Deliverer awareness (deviation bias)	Green	Green	Green
Appropriate analysis (deviation bias)	Green	Green	Green
Available data (data bias)	Green	Green	Green
Appropriate measure (measuring bias)	Green	Green	Green
Difference of measure (measuring bias)	Green	Green	Green
Assessor awareness (measuring bias)	Green	Green	Green
Pre-specified data analysis (reporting bias)	Green	Yellow	Green
Numerical analysis (reporting bias)	Green	Green	Green
Other bias	Green	Green	Green

Table S3: Results of assessment of risk bias of case-controlled and cohort studies| The Newcastle-Ottawa Quality Assessment Form was used. Green= star (low risk of bias), Yellow= unknown risk of bias, Red= No star (high risk of bias).

	**Lee *et al.*, 2005**	**Bratton *et al.*, 1999**
Representativeness of the exposed cohort (selection bias)	Green	Green
Selection of the non-exposed cohort (selection bias)	Red	Green
Ascertainment of exposure (selection bias)	Green	Green
Outcome of interest stated (selection bias)	Green	Green
Comparability bias	Red	Green
Assessment of outcome (outcome bias)	Green	Green
Appropriate follow-up of outcomes (outcome bias)	Yellow	Yellow
Appropriate follow-up of patients (outcome bias)	Green	Green
**Overall quality**	Green	Green

For peer review only

PRISMA 2020 Checklist

Section and Topic	Item #	Checklist item	Location where item is reported
**TITLE**			
Title	1	Identify the report as a systematic review.	Page 1
**ABSTRACT**			
Abstract	2	See the PRISMA 2020 for Abstracts checklist.	Page 2
**INTRODUCTION**			
Rationale	3	Describe the rationale for the review in the context of existing knowledge.	Page 3
Objectives	4	Provide an explicit statement of the objective(s) or question(s) the review addresses.	Page 3
**METHODS**			
Eligibility criteria	5	Specify the inclusion and exclusion criteria for the review and how studies were grouped for the syntheses.	Page 3
Information sources	6	Specify all databases, registers, websites, organisations, reference lists and other sources searched or consulted to identify studies. Specify the date when each source was last searched or consulted.	Page 3-4 and supplementary
Search strategy	7	Present the full search strategies for all databases, registers and websites, including any filters and limits used.	Supplementary
Selection process	8	Specify the methods used to decide whether a study met the inclusion criteria of the review, including how many reviewers screened each record and each report retrieved, whether they worked independently, and if applicable, details of automation tools used in the process.	Page 4
Data collection process	9	Specify the methods used to collect data from reports, including how many reviewers collected data from each report, whether they worked independently, any processes for obtaining or confirming data from study investigators, and if applicable, details of automation tools used in the process.	Page 4
Data items	10a	List and define all outcomes for which data were sought. Specify whether all results that were compatible with each outcome domain in each study were sought (e.g. for all measures, time points, analyses), and if not, the methods used to decide which results to collect.	Page 4
	10b	List and define all other variables for which data were sought (e.g. participant and intervention characteristics, funding sources). Describe any assumptions made about any missing or unclear information.	Page 4
Study risk of bias assessment	11	Specify the methods used to assess risk of bias in the included studies, including details of the tool(s) used, how many reviewers assessed each study and whether they worked independently, and if applicable, details of automation tools used in the process.	Page 4 and supplementary
Effect measures	12	Specify for each outcome the effect measure(s) (e.g. risk ratio, mean difference) used in the synthesis or presentation of results.	Tables
Synthesis methods	13a	Describe the processes used to decide which studies were eligible for each synthesis (e.g. tabulating the study intervention characteristics and comparing against the planned groups for each synthesis (item #5)).	Page 3-4
	13b	Describe any methods required to prepare the data for presentation or synthesis, such as handling of missing summary statistics, or data conversions.	N/A
	13c	Describe any methods used to tabulate or visually display results of individual studies and syntheses.	Page 3-4
	13d	Describe any methods used to synthesize results and provide a rationale for the choice(s). If meta-analysis was performed, describe the model(s), method(s) to identify the presence and extent of statistical heterogeneity, and software package(s) used.	Page 3-4
	13e	Describe any methods used to explore possible causes of heterogeneity among study results (e.g. subgroup analysis, meta-regression).	N/A
	13f	Describe any sensitivity analyses conducted to assess robustness of the synthesized results.	N/A
Reporting bias assessment	14	Describe any methods used to assess risk of bias due to missing results in a synthesis (arising from reporting biases).	Page 4 and supplementary
Certainty	15	Describe any methods used to assess certainty (or confidence) in the body of evidence for an outcome.	Page 4 and

For peer review only

For peer review only - http://bmjopen.bmj.com/site/about/guidelines.xhtml

PRISMA 2020 Checklist

Section and Topic	Item #	Checklist item	Location where item is reported
assessment			supplementary
**RESULTS**			
Study selection	16a	Describe the results of the search and selection process, from the number of records identified in the search to the number of studies included in the review, ideally using a flow diagram.	Figure 1
	16b	Cite studies that might appear to meet the inclusion criteria, but which were excluded, and explain why they were excluded.	N/A
Study characteristics	17	Cite each included study and present its characteristics.	Page 5-6
Risk of bias in studies	18	Present assessments of risk of bias for each included study.	Supplementary
Results of individual studies	19	For all outcomes, present, for each study: (a) summary statistics for each group (where appropriate) and (b) an effect estimate and its precision (e.g. confidence/credible interval), ideally using structured tables or plots.	N/A
Results of syntheses	20a	For each synthesis, briefly summarise the characteristics and risk of bias among contributing studies.	Page 4
	20b	Present results of all statistical syntheses conducted. If meta-analysis was done, present for each the summary estimate and its precision (e.g. confidence/credible interval) and measures of statistical heterogeneity. If comparing groups, describe the direction of the effect.	Tables
	20c	Present results of all investigations of possible causes of heterogeneity among study results.	Page 4-10
	20d	Present results of all sensitivity analyses conducted to assess the robustness of the synthesized results.	N/A
Reporting biases	21	Present assessments of risk of bias due to missing results (arising from reporting biases) for each synthesis assessed.	N/A
Certainty of evidence	22	Present assessments of certainty (or confidence) in the body of evidence for each outcome assessed.	N/A
**DISCUSSION**			
Discussion	23a	Provide a general interpretation of the results in the context of other evidence.	Page 10-11
	23b	Discuss any limitations of the evidence included in the review.	Page 10-11
	23c	Discuss any limitations of the review processes used.	Page 11
	23d	Discuss implications of the results for practice, policy, and future research.	Page 11
**OTHER INFORMATION**			
Registration and protocol	24a	Provide registration information for the review, including register name and registration number, or state that the review was not registered.	Page 3
	24b	Indicate where the review protocol can be accessed, or state that a protocol was not prepared.	Page 3
	24c	Describe and explain any amendments to information provided at registration or in the protocol.	N/A
Support	25	Describe sources of financial or non-financial support for the review, and the role of the funders or sponsors in the review.	Page 12
Competing interests	26	Declare any competing interests of review authors.	Page 12
Availability of data, code and other materials	27	Report which of the following are publicly available and where they can be found: template data collection forms; data extracted from included studies; data used for all analyses; analytic code; any other materials used in the review.	N/A

For peer review only

From: Page MJ, McKenzie JE, Bossuyt PM, Boutron I, Hoffmann TC, Mulrow CD, et al. The PRISMA 2020 statement: an updated guideline for reporting systematic reviews. BMJ 2021;372:n71. doi:

PRISMA 2020 Checklist

10.1136/bmj.n71

For more information, visit: http://www.prisma-statement.org/

For peer review only
